# The CUL5 E3 ligase complex negatively regulates central signaling pathways in CD8^+^ T cells

**DOI:** 10.1038/s41467-024-44885-0

**Published:** 2024-01-19

**Authors:** Xiaofeng Liao, Wenxue Li, Hongyue Zhou, Barani Kumar Rajendran, Ao Li, Jingjing Ren, Yi Luan, David A. Calderwood, Benjamin Turk, Wenwen Tang, Yansheng Liu, Dianqing Wu

**Affiliations:** 1 Vascular Biology and Therapeutic Program, Yale University School of Medicine, New Haven, CT 06520 USA; 2 Department of Pharmacology, Yale University School of Medicine, New Haven, CT 06520 USA; 3 Department of Dermatology, Yale University School of Medicine, New Haven, CT 06520 USA; 4 Yale Cancer Research Institute, Yale University School of Medicine, West Haven, CT 06516 USA; 5 Yale Cancer Center, Yale University School of Medicine, New Haven, CT 06520 USA

**Keywords:** Cytotoxic T cells, Cancer microenvironment, Lymphocyte activation, Neddylation

## Abstract

CD8^+^ T cells play an important role in anti-tumor immunity. Better understanding of their regulation could advance cancer immunotherapies. Here we identify, via stepwise CRISPR-based screening, that CUL5 is a negative regulator of the core signaling pathways of CD8^+^ T cells. Knocking out CUL5 in mouse CD8^+^ T cells significantly improves their tumor growth inhibiting ability, with significant proteomic alterations that broadly enhance TCR and cytokine signaling and their effector functions. Chemical inhibition of neddylation required by CUL5 activation, also enhances CD8 effector activities with CUL5 validated as a major target. Mechanistically, CUL5, which is upregulated by TCR stimulation, interacts with the SOCS-box-containing protein PCMTD2 and inhibits TCR and IL2 signaling. Additionally, CTLA4 is markedly upregulated by CUL5 knockout, and its inactivation further enhances the anti-tumor effect of CUL5 KO. These results together reveal a negative regulatory mechanism for CD8^+^ T cells and have strong translational implications in cancer immunotherapy.

## Abstract (web summary)

CD8 + T cells are central players in anti-tumour immunity. Here authors identify Cul5, a ubiquitin E3 ligase as an important inhibitor of CD8 + T cell anti-tumour cytotoxicity and persistence via involvement with both T cell receptor and cytokine-regulated central pathways.

## Introduction

CD8^+^ T cells play a central role in cancer immunotherapy. including immune checkpoint inhibition (ICI) and adaptive cell transfer (ACT). Current approved ICI in clinic relies on the re-activation of anti-tumor CD8^+^ T cells by neutralizing co-inhibitory molecules CTLA4 and/or PD1/PD-L1^1–4^. Antibodies targeting other co-inhibitory and co-stimulatory molecules such as Tim3, Lag3 and 4-1BB are being actively developed and investigated in clinical trials to further release the power of anti-tumor CD8^+^ T cells^5,6^. ACT. including tumor infiltrating lymphocytes (TILs)^7–10^, chimeric antigen receptor engineered T (CAR-T) cells^11–14^ and T cell receptor engineered T (TCR-T) cells^15–17^ also has shown promising clinical efficacy in a subset of cancer patients with malignancies otherwise refractory to other treatments. However, many hurdles still exist to prevent the successful application of ICI and ACT to the remaining majority of patients^18,19^. The immunosuppressive tumor microenvironment (TME) is one of the critical hurdles as it diminishes the effector functions and persistence of pre-existing endogenous anti-tumor CD8^+^ T cells as well as adoptively transferred T cells^20^. Genome-wide screens in T cells have been performed to identify genes that either positively or negatively regulate anti-tumor functions of cytotoxic T cells^21–27^, providing new opportunities to overcome the immunosuppressive hurdles in tumors. However, as enrichment or depletion-based screening systems are highly context-dependent, the selection pressures and criteria applied in different studies will result in the identification of differential targets, suggesting that proper selection of a clinically relevant screening pressure should result in better translation into clinical application. Among various immunosuppressive factors in TME, Transforming Growth Factor (TGF)-β appears to be a common one that directly suppresses T cell proliferation and effector functions, while promoting the exhaustion of cytotoxic T cells^28–30^. Although directly targeting TGFβ proximal signaling by overexpression of dominant negative TGFbRII on CAR-T cells revealed a promising efficacy and safety in a recent reported phase I clinical trial, TGFβ as a pleiotropic cytokine also maintains an immune homeostasis to prevent autoimmunity^31^, facilitates the formation of resident memory^32–34^ and memory precursor T cells^30^, and prevents the malignant transformation of pre-malignant cells^35^. Therefore, it is clinically important to identify other proteins either in the distal TGFβ signaling pathway or in parallel pathways that can counteract TGFβ immunosuppressive effects without systemically severe inflammatory side effects, compromising the persistence, or possibly increasing malignant transformation of adoptively transferred cytotoxic T cells.

E3 ubiquitin ligases have been shown to regulate T cell responses via the ubiquitination and subsequent degradation of TCR signaling molecules^36^. CUL5 is a key scaffold molecule in the cullin-ring E3 ligase (CRL) complex that consists of Elongin C, Elongin B, RBX2, and one of the SOCS-box-containing proteins. The SOCS-box-containing proteins act as the receptors for a specific set of substrate proteins and mediate their ubiquitination and degradation^37^. The CIS/SOCS family SOCS-box-containing proteins bind to CUL5 with variable affinities through the SOCS-box domain^38^, and some of these proteins including CISH, SOCS1 and SOCS3 have been shown to play regulatory roles in T cell activation by targeting cytokine and TCR signaling^39,40^. However, it is not known whether the CUL5 E3 ligase complexes regulate CD8^+^ T cell activation or function through any of these CIS/SOCS family proteins in CD8^+^ T cells despite CUL5 regulates phosphorylated JAK1 degradation by interacting with CISH in CD4 cells^41^. Neddylation is a critical modification of CRLs to induce their conformational changes and subsequent activation^42^. As overactivated neddylation of CRLs is associated to disease progression and poor survival of multiple human cancers^43–45^, neddylation inhibitors blocking E1 NEDD8-activating enzyme such as MLN4924 and TAS4664 have been developed and investigated in over 40 clinical trials (https://www.clinicaltrials.gov/) to evaluate their safety and anti-tumor efficacy. Besides its effects on tumor cells, neddylation has been shown to regulate the functions of various immune cells in TME including CD4^+^ T cells^46,47^. In addition, neddylation of cullins 1-4 but not cullin 5 through Ube2m or Rbx1 showed its critical role in the maintenance of Treg cell fitness^48^. Therefore, knockout of *Ube2m* or *Rbx1* in mice developed an early-onset fatal inflammatory disorder associated with dysfunctional Tregs. However, little is known about the effect of neddylation on CD8^+^ T cells.

In this study, we developed a stepwise CRISPR loss of function screening approach by performing whole-genome in vitro screens in the presence of TGFβ and targeted in vivo bulk screens, followed by in vivo single-cell screens. This approach allows us to robustly identify gene targets, including CUL5, in CD8^+^ T cells that regulate anti-tumor efficacy. Proteomic analyses and biochemical studies indicate that CUL5 interacts with an understudied SOCS-box-containing substrate receptor protein, PCMTD2 and negatively regulates TCR and IL2 signaling in CD8 T cells.

## Results

### Stepwise bulk CRISPR KO screens enrich potential ACT boosters

**Fig. 1:**
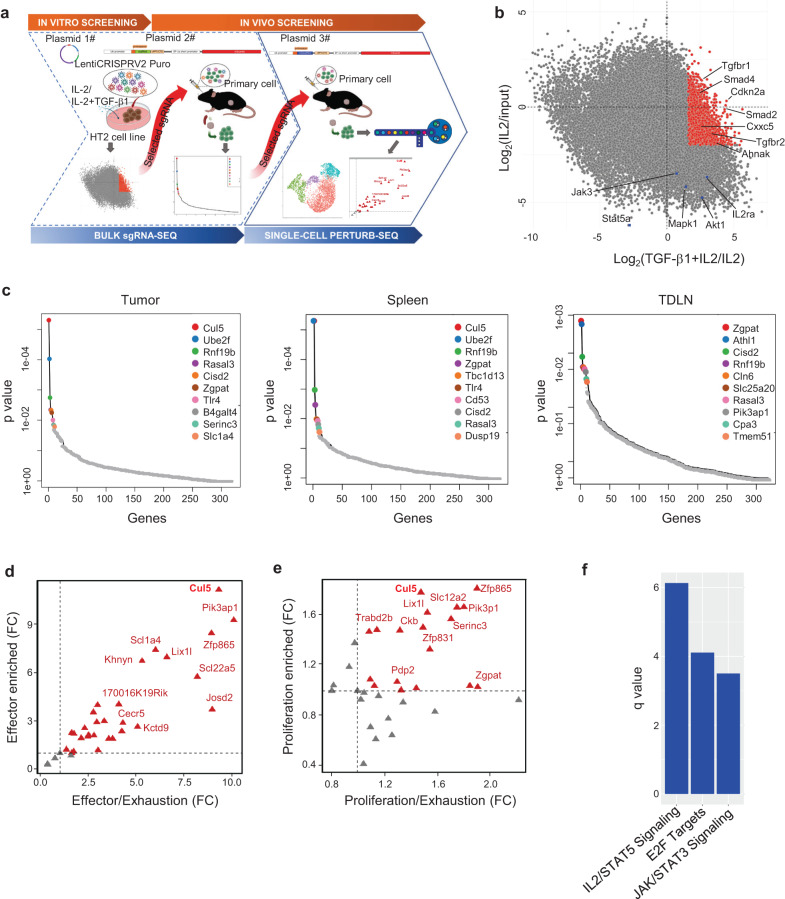
Step-wise CRISPR KO screens identify genes enhancing anti-tumor activity of CD8^+^ T cells. **a** Schematic representation of the experimental design for step-wise CRISPR KO screens. The icons are created with BioRender.com. **b** Scatter plots of sgRNA log_2_-fold change (x-axis, TGFβ1 + IL2/IL2; y-axis, IL2/input) in HT2 cells cultured in vitro for 21 days. **c** Gene enrichment analysis of the bulk in vivo screen in tumors (Left), spleens (Middle), and TDLNs (Right) using the MAGeCK analysis. **d, e** Genes enriched in the **d**, effector cluster and **e**, proliferating cluster in the single cell CRISPR KO screening are presented as the target gene enrichment over the non-targeting control being plotted against the ratio of the enrichment in **d**, effector cluster or **e**, proliferating cluster to that in the exhausted cluster. **f** Signaling pathways enriched in transferred tumor-infiltrating Cas9/OT-I cells expressing *Cul5* sgRNAs compared to those expressing non-targeting sgRNAs.

To identify genes that may provide TGFβ resistance in the context of CD8 T cells, we developed a stepwise CRISPR screening approach combining an initial T cell line-based in vitro genome-wide screening with primary CD8^+^ T cell in vivo screens at the bulk and then single cell levels (Fig. 1a). This design allowed us to circumvent the cost and labor ineffectiveness of performing a genome-wide in vivo CRISPR/Cas9 screen, which, due to library size and the very limited number of tumor-infiltrating T cells^21,24^, would require a large number of mice. IL2 is important for T cell proliferation and function in vivo^49^ and for generation of adoptive transferred T cell products in vitro^50^, while TGFβ is a common and dominant immunosuppressive factor in TME^51^. Therefore, for the initial genome-wide CRISPR screen, we used an immortal mouse T cell line, HT2, whose proliferation is IL2 dependent^52^ and suppressed by TGFβ^53^. The HT2 T cells were transduced with the Briea genome-wide mouse CRISPR KO library in a lentiviral vector co-expressing spCas9 and sgRNA^54^. As shown in Supplementary Fig. 1b, compared to IL2 culture alone, the addition of TGFβ1 significantly but not completely suppressed the proliferation of CRISPR library transduced HT2 cells. The library-infected HT2 cells that were immediately after antibiotic selection were collected and subjected for the next generation sequencing (NGS), which is designated as the input. After 21 days of culture in the presence IL2 or IL2 + TGFβ1, the cells were subjected to NGS and designated as IL2 and IL2 + TGFβ1. Gene depletion and enrichment between IL2 and input and between IL2 + TGFβ1 and IL2 were analyzed by MAGeCK and are plotted in Fig. 1b. Among the significantly depleted genes of NGS_IL2_ vs NGS_Input_ are many known for IL2 signaling: *Akt1, Eras, Grb2, Il2ra, Il2rb, Jak3, Mapk1, Pik3ca, Pik3cb, Ptpn11, Raf1, Shc1, Sos1, Stat5a, and Stat5b*. Among the significantly enriched genes of NGS_IL2+TGFβ1_ vs NGS_IL2_ are known TGFβ signaling genes: *Smad1, Smad2, Smad4, Tgfb3, Tgfbr1, Tgfbr2 and Rnf111*. As a validation for the in vitro screen, we performed Ingenuity Pathway Analysis of significantly (*P* < 0.05 by MAGeCK) depleted genes of IL2 vs input and enriched genes of IL2 + TGFβ1 vs IL2. The most affected pathways in the IL2 vs input analysis were basic cellular processes, including metabolism, translation, transcription, cell cycle, and DNA repair. Protein ubiquitination, IL2 signaling, and those related to IL-2 signaling, including the PI3K, JAK, mTOR pathways were also significantly depleted (Supplementary Fig. 1c and Supplementary Data 1). Among the significantly enriched genes upon TGFβ1 treatment are the TGFβ-signaling as well as several other processes (EMT, Senescence, and adherens junction) that TGFβ signaling is known to be involved in (Supplementary Fig. 1c and Supplementary Data 1). These results suggest that the in vitro screen is sufficiently robust. Given our primary objective to find genes that may provide resistance to TGFβ, we used the following four criteria to select putative candidate genes for the in vivo screen: 1) They should not interrupt IL2-dependent cell survival and proliferation, which means genes depleted in Day-21 IL2 culture alone compared to pre-culture input should be excluded from consideration; 2) They should resist TGFβ1-dependent suppression of proliferation, which means genes enriched in Day-21 IL2 + TGFβ1 culture compared to Day-21 IL2 culture alone should be included in consideration; 3) The candidate genes should have detectable transcription levels in primary CD8^+^ T cells either at resting state or after activation based on the Immgen dataset^55^; and 4) the genes already known related to TGFβ should be excluded as we intended to identify genes that provide TGFβ resistance. Finally, 256 candidate genes were selected for further in vivo screens in primary CD8^+^ T cells (Supplementary Data 1).

We used OT-I T cells against ovalbumin (OVA)-expressing tumor as an in vivo ACT model to perform the secondary CRISPR screening. We compared TGFβ expression levels among four commonly used C57Bl mouse syngeneic cancer cell lines and found that EL4 expressed the highest level of TGFβ (Supplementary Fig. 1d). Thus, we chose the corresponding OVA-expressing EL4 cell line E.G7-OVA for further screens to ensure the presence of enhanced TGFβ immunosuppressive microenvironment. We generated Cas9-expressing OT-I (Cas9/OT-I) T cells by crossing OT-I and Cas9 mice. Cas9 and OT-I TCR expression in CD8^+^ T cells were confirmed by flow cytometry (Supplementary Fig. 1e), and OVA antigen-specific T cell activation was validated by degranulation and cytokine production after co-culture with E.G7-OVA cells (Supplementary Fig. 1f). Gene knockout efficiency over 70% of Cas9/OT-I T cells was confirmed with CD8a KO by retroviral transduction of an sgRNA targeting CD8a (Supplementary Fig. 1g). A customized sgRNA library containing sgRNAs (six for each target gene) targeting the 270 selected genes was generated with the coverage and normal distribution of sgRNAs confirmed by next generation sequencing (Supplementary Fig. 1h). Library-transduced Cas9/OT-I T cells were sorted based on the mScarlet reporter expression and then transferred into E.G7-OVA tumor-bearing mice. Upon T cell transfer, tumors shrunk rapidly in the first 7 days and then became relatively stable for the next 5 days (Supplementary Fig. 1i), suggesting a switch of T cell anti-tumor immune response from an active effector stage to an immune suppression/exhaustion stage as previously reported^56^. Because tumors can also suppress immune responses systemically^57,58^, library-transduced T cells were collected from tumors, tumor-draining lymph nodes (TDLNs), and spleens by sorting CD8^+^GFP^+^ cells at day 12 post T cell transfer. We reasoned that the proliferation and persistence of immune suppression-resistant T cells should outcompete others, which can be reflected by the enrichment of sgRNAs in the library. Therefore, sgRNA enrichment analysis was carried out by comparing each sample with the pre-transfer input. As the step-wise screening significantly limited in vivo sgRNA pool, an average of 150x coverage per sgRNA per tissue was achieved from recovered T cells of one recipient. A total of 4 replicates for each tissue were then used for robust sgRNA enrichment analysis. Figure 1c showed top enriched gene hits from the tumor, spleen and TDLN, respectively. *Cul5, Ube2f* and *Rnf19b* were top three hits in both tumor and spleen, while *Zgpat, Athl1* and *Cisd2* were top three hits in TDLN. Interestingly, CUL5 and UBE2F are the components of the CUL5-E3 ligase complex, suggesting a possible important role of the E3 ligase complex in the regulation of CD8^+^ T cell anti-tumor responses.

### Single-cell CRISPR screening identifies *Cul5* as a CD8 cell regulator

The above two CRISPR screens are based on an overall T cell number enrichment, which reflects the proliferation and persistence of T cells as an entire population. However, tumor-infiltrating T cells are known to have a variety of differentiation status and subpopulations^59,60^, complicating the interpretation of the bulk CRISPR screen results. Moreover, a superior anti-tumor cytotoxic T-cell response also requires enhanced cytotoxic effector functions besides the increase of total cell number^21,25^. Previous effector-based CRISPR screens are biased to a single phenotype such as degranulation (surface CD107a^+^)^21^ or cytokine production (IL2 or IFNg intracellular staining)^61^, which may not be able to completely reflect functional T cell subsets. Therefore, to comprehensively identify gene candidates regulating functional CD8^+^ T cell subgroups, we decided to perform single-cell CRISPR KO screening with tumor-infiltrating CD8^+^ T cells by single-cell RNA sequencing in parallel with single-cell sgRNA sequencing. To make our CRISPR system compatible with 10x genomics kit, the original sgRNA scaffold sequence was replaced by the one containing 10x genomics capture sequence in the stem loop region. Gene editing efficiency as high as over 80% with the new scaffold replacement was confirmed by *Cd8a* KO in primary Cas9/OT-I T cells (Supplementary Fig. 2a). Thirty-three candidate genes with 3 top efficient sgRNAs per gene plus 4 non-targeting sgRNAs were selected from the in vivo bulk screening with a selection criteria of more than one sgRNA enrichment over 1.4 fold per gene in each sample (Supplementary Data 2). Seven days post-ACT in the E.G7-OVA model, over 10,000 sorted tumor-infiltrating Cas9/OT-I T cells were subjected to 10x single-cell RNA sequencing to obtain approximate 300x coverage per target gene. Unsupervised clustering divided the cells into four clusters that were visualized in 2D Uniform Manifold Approximation and Projection (UMAP) (Supplementary Fig. 2d). Among differential expressed gene (DEG) set for each cluster (Supplementary Fig. 2b), T cell phenotype markers were used to annotate the four clusters into exhausted-like cells (*Ccl3, Ccl4, Tnfrsf9, Ifng, Lag3* and *Harvcr2*) as Cluster 0; proliferating cells (Mik67 and Cdk1) as Cluster 1; resting precursor-like cells (*Il7r, S1pr1, Tcf7* and *Slamf6*) as Cluster 2; effector cells (*Gzmc, Gzmd, Gzme, Gzmf, Prf1, Spp1, Klre1* and *Klrd1*) as Cluster 3 (Supplementary Fig. 2c)^60^. UMAP cell cycle phase analysis also showed that the majority of the cells in the G2M and S stages were in cluster 1, whereas the other three clusters were mainly in the G1 stage (Supplementary Fig. 2e), consistent with the proliferating cell identity for Cluster 1. As the exhaustion-like cluster is the unfavorable group in anti-tumor responses of cytotoxic T cells, we evaluated the enrichment of individual gene KO T cells in the other three functional clusters based on both the absolute cell counts and respective ratio to the exhaustion cluster, with the pre-transfer input and non-targeting sgRNA transduced T cells as the normalization control (Fig. 1d, e and Supplementary Fig. 2f). This systemic and unbiased functional analysis of the screen revealed that CUL5 KO cells were highly enriched in both effector and proliferating clusters. Several other hits, such as *Pik3ap1, Zfp865* and *Lix1l*, were also enriched in two or three of these functional clusters.

### CUL5 KO enhances anti-tumor effects of primary CD8^+^ T cells in vivo

In the above single cell analysis, the effector cluster showed much higher enrichment magnitude compared to the proliferating and precursor-like clusters (Fig. 1d, e and Supplementary Fig. 2f), suggesting the stronger impact of gene perturbation on the effector population. Among top enriched gene (*Pik3ap1, Cul5* and *Zf865*) KO in the effector cluster, the role of PIK3AP1 in CD8^+^ T cell activation through PI3K signaling has been reported^62^, while *Zf865* is a mouse specific gene. CUL5 as a core element of E3 ligase complexes, on the other hand, has not been investigated in CD8^+^ T cells, despite a recent study reporting its regulation of CD4^+^ T helper cell differentiation^41^. Therefore, we decided to investigate CUL5 further. The fact that the *Cul5* gene expression level was much lower in the CUL5 KO cells than non-targeting KO cells or other gene KO cells (Supplementary Fig. 2g) confirms that the *Cul5* sgRNAs yielded a high KO efficiency. A further gene enrichment and signaling pathway analysis of DEG between the *Cul5* KO and non-targeting KO cells indicated that the IL2/STAT5, E2F targets and JAK/STAT3 signaling pathways were significantly altered (Fig. 1f). Taken together, our step-wise CRISPR KO screens revealed CUL5 may play an important role in regulating the persistence and effector functions of tumor-reactive CD8^+^ T cells.

**Fig. 2:**
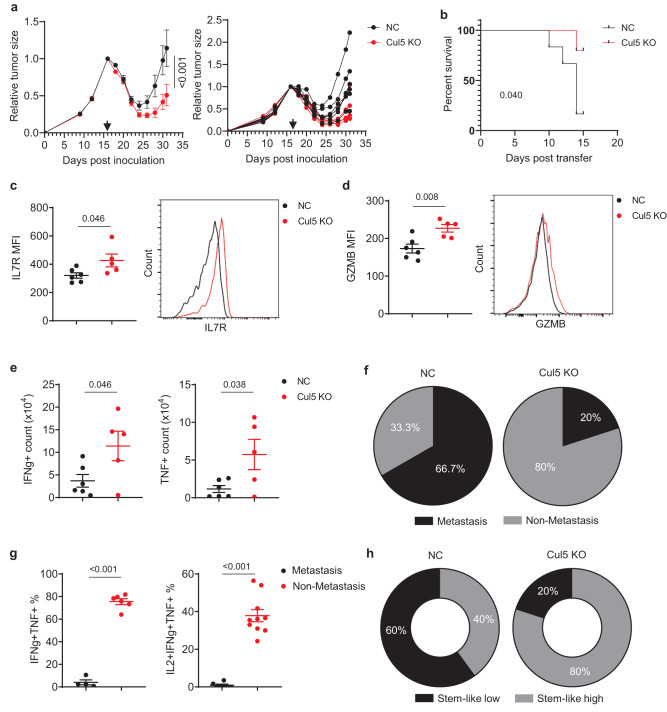
CUL5 KO in primary CD8^+^ T cells enhanced anti-tumor responses. **a** The effect of the transfer of CUL5 KO or non-targeting (NC) OT-I CD8 + T cell on tumor progression was examined in the C57BL/6 N mice inoculated with the E.G7-OVA cells. Data are shown as mean ± SEM on the left and as individual mouse on the right. Black arrow indicates the time of sub-lethal irradiation followed by immediate adoptive transfer of CUL5 (Red) KO or NC (Black) Cas9/OT-I cells. Two-way ANOVA (Sidak correction), *n* = 5 CUL5 KO and 6 NC mice per group). **b**, Survival curve of E.G7-OVA tumor-bearing C57BL/6 N mice post adoptive transfer of CUL5 KO (Red) or NC (Black) Cas9/OT-I cells. Gehan-Breslow-Wilcoxon test (*n* = 5 mice per group). **c, d** Flow cytometry analysis of **c**, CD127 and **d** Granzyme B expression of transferred tumor-infiltrating CUL5 KO or NC Cas9/OT-I cells. Representative histograms are shown. Data are shown as mean ± SEM (Two-sided unpaired t-test, *n* = 5 CUL5 KO and 6 NC mice). **e** Flow cytometry analysis of IFNg^+^ (Left) and TNF^+^ (Right) cell counts per tumor of transferred tumor-infiltrating Cas9/OT-I cells post re-stimulation in vitro. Data are shown as mean ± SEM (Two-sided unpaired t-test; *n* = 5 CUL5 KO and 6 NC mice). **f** Pie chart showing the percentage of lymph node (LN) metastasis (Black) and non-metastasis (Grey) in each group. **g** Flow cytometry analysis of IFNg^+^TNFa^+^ (Left) or IL2^+^IFNg^+^TNFa^+^ (Right) transferred TDLN-infiltrating Cas9/OT-I cells post re-stimulation in vitro in LNs with (Black) and without metastasis (Red). Data are shown as mean ± SEM (Two-sided unpaired t-test; *n* = 6 or 13 CUL5 KO and 4 or 6 NC mice). **h** Donut chart showing the percentage of exhaustion subtype (Black)-dominant and progenitor subtype (Grey)-dominant transferred TDLN-infiltrating Cas9/OT-I cells in each group.

To validate the enhanced anti-tumor responses of CUL5 KO CD8^+^ T cells, Cas9/OT-I T cells transduced with the *Cul5* sgRNAs or a non-targeting control sgRNA were adoptively transferred into E.G7-OVA tumor-bearing mice subjected to prior sub-lethal irradiation for lymphodepletion^63^. The CUL5 KO T cells showed significantly stronger tumor control ability than the NC T cells (Fig. 2a), resulting in significantly improved survival (Fig. 2b and Supplementary Fig. 3a). Further analysis of tumor-infiltrating transferred T cells as well as those in TDLNs at the end point revealed that the CUL5 KO T cells had significantly higher IL7R (Fig. 2c and Supplementary Fig. 3b) and GZMB (Fig. 2d and Supplementary Fig. 3c) expression levels than the NC cells. These results suggest that the CUL5 KO T cells possess a hybrid stemness/effector phenotype, which may explain their improved functional persistence. Concordantly, the CUL5 KO group had a significantly higher number of tumor-infiltrating transferred T cells that were still capable of producing IFNg and/or TNFa upon in vitro re-stimulation than the NC group (Fig. 2e, Supplementary Fig. 3d, e). Other tumor-infiltrated immune cell types, including Treg cells, macrophages and NK cells showed little differences between CUL5 KO and NC (Supplementary Fig. 3f), suggesting the improved therapeutic effect of CUL5 KO CD8^+^ T cells is largely due to transferred T cells. In addition, transfer of the CUL5 KO T cells reduced the incidence of TDLN-metastasis of primary E.G7-OVA tumors from 65% to 20% (Fig. 2f), suggesting that the CUL5 KO T cells may suppress metastasis along with a better control of primary tumors. TDLN has been demonstrated as a reservoir of tumor-specific stem-like CD8^+^ T cells^64^ that preserve the ability of multiple cytokine-producing ability upon re-stimulation^65^ and are important for ICI efficacy and sustained anti-tumor responses^66^. In this study, we observed a negative correlation of the proportion of transferred stem-like CD8^+^ T cells in TDLN capable of producing multiple cytokines with TDLN metastasis (Fig. 2g and Supplementary Fig. 3g) regardless of CUL5 KO. This observation suggests that established metastatic tumor cells may induce exhaustion of anti-tumor stem-like CD8^+^ T cells in TDLN, which is consistent with a similar finding in a clinical breast cancer metastasis study^67^. Concordantly to the reduced metastasis incidence, we also observed an increased incidence of TDLN possessing high proportion of stem-like CD8^+^ T cells (multiple cytokine producer upon restimulation, from 40% to 80%) in the CUL5 KO group (Fig. 2h), suggesting a better preservation of functional stem-like CD8^+^ cells with CUL5 KO. These results together indicate that CUL5 KO in T cells have an increased hybrid stemness/effector phenotype that enhances their anti-tumor effects in both primary tumor suppression and metastasis prevention in vivo.

### CUL5 KO enhances effector activities of primary CD8^+^ T cells in vitro

**Fig. 3:**
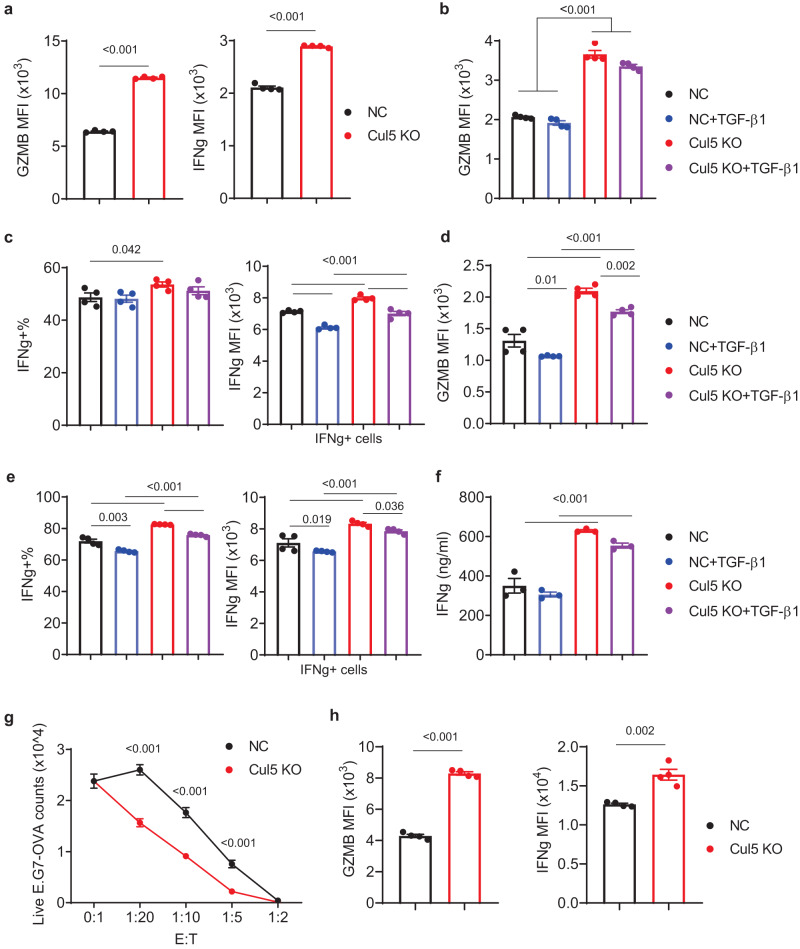
Immunological characterization of CUL5 KO primary CD8^+^ T cells in vitro. **a**–**e** Flow cytometry analysis of effector molecules (GZMB and IFNg) in NC (Black and Blue) or CUL5 KO (Red and Purple) primary CD8^+^ T cells **a**, prior to or post anti-CD3+anti-CD28 stimulation for **b, c**, 6 h or **d, e**, 16 h in the presence (Blue and Purple) or absence (Black and Red) TGFβ1. Data are shown as mean ± SEM (Two-sided unpaired t-test, *n* = 4). **f** ELISA analysis of secreted IFNg from NC (Black and Blue) or CUL5 KO (Red and Purple) primary CD8^+^ T cells 16 h post anti-CD3+anti-CD28 stimulation in the presence (Blue and Purple) or absence (Black and Red) TGFβ1. Data are shown as mean ± SEM (Two-sided unpaired t-test, *n* = 3). **g**, In vitro cytotoxicity assay of NC (Black) or CUL5 (Red) KO OT-I cells co-cultured with E.G7-OVA cells overnight. E:T, effector (T cell) to target (tumor cell) ratios. Data are shown as mean ± SEM (Two-sided two-way Anova with sidak correction, *n* = 4). **h** Flow cytometry analysis of GZMB and IFNg expression of NC (Black) or CUL5 KO (Red) OT-I cells co-cultured with E.G7-OVA cells at a E:T ratio of 1:20 for 6 h. Data are shown as mean ± SEM (Two-sided unpaired t-test, *n* = 4). Datum points represent biological replicates.

To further investigate the effects of CUL5 KO on primary CD8 T cell responses, we generated CUL5 KO CD8^+^ T cells using cells isolated from the spleens of the Cas9/OT-I mice, and performed cytokine-induced differentiation, TCR-dependent activation, and tumor cell killing assays in vitro. Continued differentiation and expansion of CUL5 KO T cells in the IL2, IL7 and IL15 cytokine cocktail prior to TCR-dependent stimulation already showed increased intracellular GZMB and IFNg expression compared to the NC T cells (Fig. 3a and Supplementary Fig. 3h), suggesting that CUL5 is actively involved in cytokine-dependent cytotoxic T cell differentiation. We then examined multiple effector markers using flow cytometry 6 and 16 h post anti-CD3 stimulation in the presence or absence of TGFβ1. GZMB and IFNg were significantly higher at both time points in the CUL5 KO cells than the NC ones (Fig. 3b–e, Supplementary Fig. 3i–l). In agreement with flow cytometry results, secreted IFNg detected by ELISA was significantly higher in CUL5 KO cells than the NC cells as well (Fig. 3f). GZMB expression at 16 h and IFNg expression at both 6 h and 16 h were significantly suppressed by TGFβ1 to similar degrees in CUL5 KO and NC cells (Fig. 3c–e), suggesting that CUL5 may be not directly involved into TGFβ signaling and cannot be completely suppressed by TGFβ. Because Gzmb expression (Fig. 3b, d) and IFNg production (Fig. 3e, f) in the CUL5 KO cells in the presence of TGFβ1 were still significantly higher than those in its absence, CUL5 KO would present an apparent TGFβ1 resistance. When co-cultured with the E.G7-OVA cells, in vitro differentiated OT-I T cells with CUL5 KO showed significantly higher tumor cell killing ability at several non-saturated effector to target ratios than the NC cells (Fig. 3g), accompanied with significantly increased GZMB and IFNg expression levels upon E.G7-OVA cell stimulation over those of the NC cells (Fig. 3h and Supplementary Fig. 3m). These data together connect CUL5 to the regulation of cytotoxic T cell differentiation, activation, and effector functions.

### CUL5 KO alters CD8^+^ T cell proteome

**Fig. 4:**
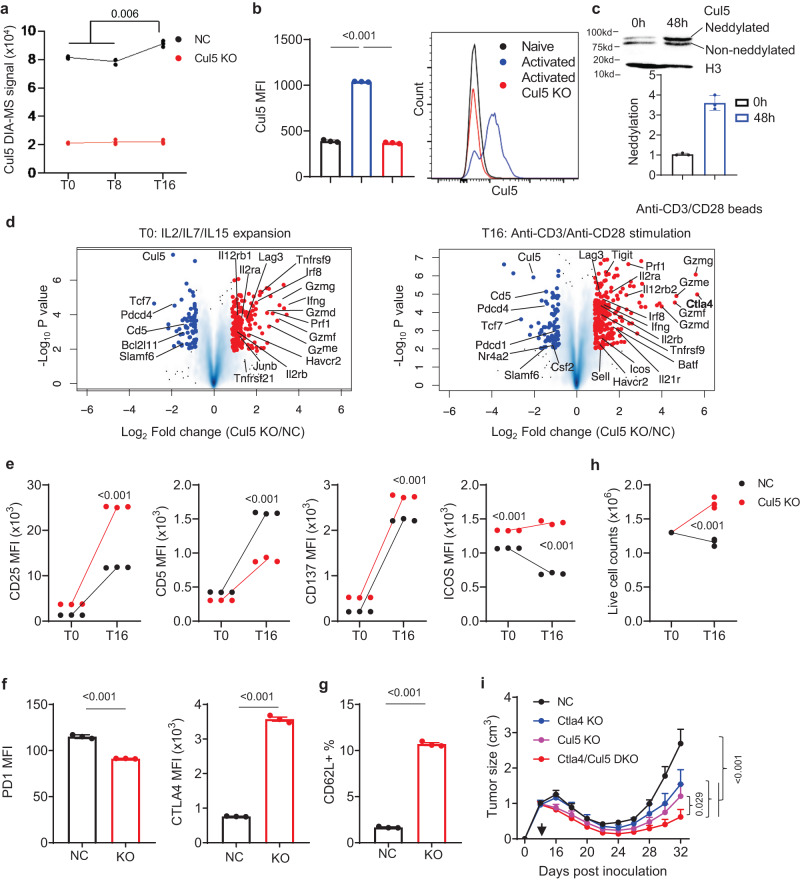
CUL5 KO causes proteomic alterations in primary CD8^+^ T cells. **a** DIA-MS signals of the CUL5 protein in NC (Black) or CUL5 (Red) KO primary CD8^+^ T cells at the T0, T8 and T16 conditions determined by mass spectrometry (MS). Data are shown as mean ± SEM (Two-sided multiple t-test, *n* = 3). **b** Flow cytometry analysis of CUL5 expression in primary CD8^+^ T cells before (Black) or after (Blue) 2-day activation by anti-CD3/CD28 beads, with 2-day activated CUL5 KO cells as a control (Red). Data are shown as mean ± SEM (Two-sided unpaired t-test, *n* = 3). **c** Western blot analysis of CUL5 expression in primary CD8^+^ T cells treated as in **b**. Neddylated CUL5 (Bottom) was quantified, with Histone H3 as internal control. **d** Volcano plot of differentially expressed proteins between CUL5 KO and non-targeting (NC) primary CD8^+^ T cells at T0 (Left) and T16 (Right) conditions as quantified by DIA-MS (*n* = 3). **e**–**g** Flow cytometry validation of immunological markers (CD25, CD5, CD137, ICOS, PD1, CTLA4 and CD62L) altered in CUL5 KO primary CD8^+^ T cells at T0 (**e**) and/or T16 (**e**–**g**) conditions. Data are shown as mean ± SEM (Two-sided unpaired t-test, *n* = 3). **h** Live cell numbers of NC (Black) and CUL5 (Red) KO primary CD8^+^ T cells before and after 16-hour anti-CD3 plus anti-CD28 stimulation. Data are shown as mean ± SEM (Two-sided unpaired t-test, *n* = 3). **i** Growth curve of tumors from E.G7-OVA cells inoculated s.c. into C57BL/6 mice. Data are shown as mean ± SEM. The back arrow indicates the time of sub-lethal irradiation followed by immediate adoptive transfer of Cas9/OT-I cells with NC (Black), CTLA4 (Blue), CUL5 (Pink) and CTLA4/CUL5 double (Red) KO. (Two-way ANOVA with Tukey correction; *n* = 4–5). Datum points represent biological replicates.

To investigate the mechanism by which CUL5 regulates CD8^+^ T cell cytokine-dependent differentiation and TCR-dependent activation, we performed quantitative mass spectrometry (MS) by data-independent acquisition (DIA)-MS^68^ of total proteins in the CUL5 KO and NC primary CD8^+^ T cells in the following conditions: 1) Cytokine dependent expansion and differentiation (T0); 2) 8 h cytokine withdraw prior to TCR stimulation (T8); and 3) 16 h TCR stimulation post 8-hour cytokine withdraw (T16). Principal component analysis (PCA) and correlation analysis revealed that replicates in each condition clustered together while different conditions separated from each other (Supplementary Fig. 4a,b), suggesting significant proteomic changes among different conditions of the same T cells as well as between CUL5 KO and NC T cells in each condition. Together with similar total protein quantities among all detected samples (Supplementary Fig. 4c), DIA-MS analyses were of high quality. Additionally, the strong reductions of CUL5 abundances in the CUL5 KO cells from all three conditions compared to the NC cells (Fig. 4a) confirms high KO efficiency. Of note, the proteomic analysis showed that the CUL5 protein level was significantly increased upon TCR stimulation post cytokine starvation in the NC cells, suggesting a potential negative feedback regulatory role of CUL5 in CD8^+^ T cell activation (Fig. 4a). Consistent with this idea, we observed more markedly upregulation of the CUL5 protein upon of TCR stimulation of naïve primary CD8^+^ cells than TCR restimulation (Fig. 4b). More importantly, TCR stimulation also increased neddylated form^42,69^ of CUL5 in these primary CD8^+^ T cells (Fig. 4c). Upon differential expression analysis of the proteomic data with stringent cutoffs (*p* value < 0.01 and FC > 1.8 or <0.55), we found 184 and 69 in T0; 177 and 65 in T8; 234 and 79 in T16 up- and down-regulated proteins, respectively, in the CUL5 KO T cells compared to NC T cells (Supplementary Data 3 and Fig. 4d). The commonly and uniquely regulated proteins among three conditions are shown in the Venn diagram (Supplementary Fig. 4d and Supplementary Data 4). Cytotoxic effectors like granzymes, perforin and IFNg were commonly upregulated, while the negative regulators of effector responses such as TCF7, SLAMF6, PDCD4 and CD5 were commonly downregulated^70,71^ (Fig. 4d). Several other functional proteins including TCR/cytokine signaling (CD247, CD3e, CD3d, CD3g, TCRA, CD28, IL12RB1/2, IL17RA, IL2RA/B, IFNAR1, IL21R, JAK3 and STAT3), stimulatory or inhibitory checkpoint molecules (TNFRSF21, CTLA4, ICOS, TIGIT, TNFRSF18, HAVCR2, LAG3, TNFRSF8 and TNFRSF9), and transcription factors for T cell activation (JUN, JUNB, FOSL2, IRF8 and BATF) increased in at least one condition (Fig. 4d and Supplementary Data 4). Of note, several negative regulators of anti-tumor responses were down-regulated in CUL5 KO cells including exhaustion-associated biomarkers (NR4A2, PDCD1 and CD160), myeloid-derived suppressive cell promoting cytokine CSF2, and pro-apoptosis factor BCL2L11. Although TCF7 as a key transcription factor for memory cell formation reduced in CUL5 KO cells, SELL as another marker of memory precursor increased significantly upon TCR stimulation compared to NC cells^72^. Flow cytometry was performed to confirm the MS results on several critical markers of tumor immune responses including CD25 (IL2RA), CD5, CD137 (TNFRSF9), ICOS, PD1 (PDCD1), CTLA4, and CD62L (SELL) (Fig. 4e–g). In addition, after 16-hour stimulation, the number of live CUL5 KO cells increased, whereas that of NC cells did not, compared to pre-stimulation (Fig. 4h). These results together are consistent with the overall enhanced persistence and cytotoxic activity of CUL5 KO T cells.

We also noticed that several co-inhibitory checkpoint molecules increased along with the enhanced anti-tumor functions of CUL5 KO cells. These changes may reflect negative feedback regulation as the results of elevated T cell activation in the CUL5 KO cells. Among these molecules, the CTLA4 protein content (Fig. 4d, f) as well as *Ctla4* mRNA (Supplementary fig. 4g) showed the strongest increase in CUL5 KO cells. Therefore, we reasoned that knockout of CTLA4 in combination of CUL5 KO may further release the cytotoxic power of anti-tumor CD8^+^ T cells. To this end, in the same E.G7-OVA tumor model, we compared the anti-tumor effects of NC, CTLA4 KO, CUL5 KO and CTLA4/CUL5 double KO (DKO) OT-I T cells post adoptive transfer. Ctla4 KO alone showed similar ability in tumor control to CUL5 KO. However, as anticipated, CTLA4 and CUL5 DKO showed a superior anti-tumor ability compared to either CTLA4 KO or CUL5 KO alone (Fig. 4i and Supplementary Fig. 4e). This result suggests a therapeutic potential of ACT with a combinatory CUL5 and CTLA4 knockout.

### CUL5 interacts with PCMTD2 and targets TCR/IL2 signaling

**Fig. 5:**
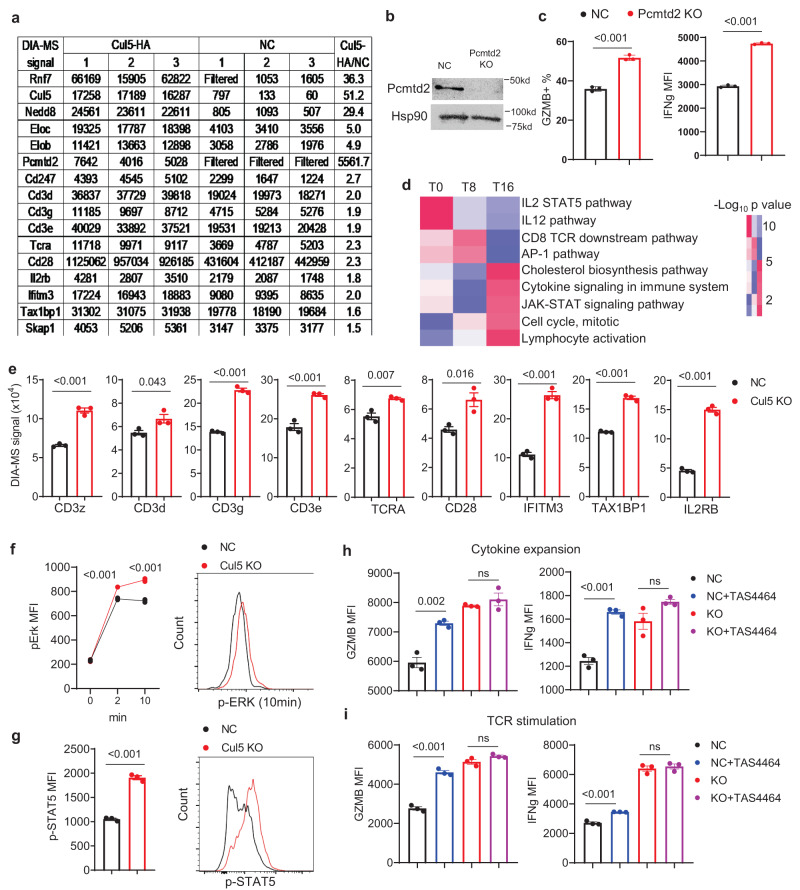
The CUL5 E3 complex targets TCR and IL2 signaling in CD8^+^ T cells. **a** DIA-MS signals of enriched proteins in TCR-activated mouse primary CD8^+^ T cells transduced with CUL5-HA overexpressing vector (CUL5-HA) vs empty control vector (NC) identified by anti-HA co-IP MS (Cut-off: *p* value < 0.05 and fold change >1.5 in multiple t-test). **b** Western blot analysis of PCMTD2 expression in primary mouse CD8^+^ T cells with NC or PCMTD2 KO. HSP90 as an internal control. This analysis was repeated three times. **c**, Flow cytometry analysis of GZMB (as positive % in cytokine expansion condition, Left) and IFNg (as MFI in 6-hour anti-CD3/CD28 stimulation condition, Right) expression of NC (Black) or PCMTD2 KO (Red) primary mouse CD8^+^ T cells. Data are shown as mean + SEM (Two-sided unpaired t-test, *n* = 3). **d** Heatmap of signaling pathway enrichment of CUL5 KO CD8^+^ T cells compared to NC cells based on differentially expressed proteins using DIA-MS quantification. **e** DIA-MS signals of proteins identified by the total protein MS analysis of mouse primary CUL5 KO (Red) and NC (Black) CD8^+^ T cells post TCR stimulation. Data are shown as mean ± SEM (Two-sided unpaired t-test, *n* = 3). **f** Flow cytometry analysis of p-ERK1/2 expression in NC or CUL5 KO primary mouse CD8^+^ T cells after anti-CD3 plus anti-CD28 stimulation. Data are presented (Left) as mean ± SEM (Two-sided unpaired t-test, *n* = 3). **g** Flow cytometry analysis of p-STAT5 expression in NC or CUL5 KO primary mouse CD8^+^ T cells after 16-hour anti-CD3 plus anti-CD28 stimulation supplied with 5 ng/ml hIL2. Data are presented (Left) as mean ± SEM (Two-sided unpaired t-test, *n* = 3). **h**, **i**, Flow cytometry analysis of GZMB and IFNg in NC (Black and Blue) or CUL5 KO (Red and Purple) primary CD8^+^ T cells in 16-hour **h**, cytokine culture (IL2/IL7/IL15) or **i**, anti-CD3 plus anti-CD28 stimulation with (Blue and Purple) or without (Black and Red) 250 nM TAS4464. Data are shown as mean ± SEM (ns, not significant; two-sided unpaired t-test, *n* = 3). Datum points represent biological replicates.

To identify CUL5 interacting proteins in CD8^+^ T cells, we overexpressed CUL5 with a C terminal HA-tag (CUL5-HA) in mouse primary CD8^+^ T cells by retroviral transduction. The cells were subjected to TCR stimulation for 12 h, and CUL5-HA was immunoprecipitated by anti-HA, followed by DIA-MS analysis (co-IP-MS). Compared to the negative control samples (cells transduced with the empty vector), 65 proteins were enriched (*p* value < 0.05 and Log2fold change >0.66) in the anti-HA IP samples (Supplementary Data 5). CUL5 and the obligate CUL5 E3 ligase complex subunits including RNF7, NEDDY8, ELOB and ELOC were highly enriched (Fig. 5a). Among all of the known SOCS-box-containing proteins that have the potential to interact with CUL5 and were detectable in the total protein MS analysis (Fig. 5b), Pcmtd2^73^ is the only one enriched in the CUL5-HA IP samples (Fig. 5a). The interaction between exogenously expressed and endogenous CUL5 and PCMTD2 was subsequently confirmed by co-immunoprecipitation (Supplementary Fig. 5a, b). To demonstrate the importance of PCMTD2 in TCR/cytokine signaling, we performed PCMTD2 KO in mouse primary CD8^+^ T cells followed by cytokine-induced expansion/differentiation and TCR-induced re-stimulation. PCMTD2 KO efficiency was confirmed by Western blotting analysis (Fig. 5b). Consistent to CUL5 KO, the expression of GZMB and IFNg was significantly higher in PCMTD2 KO CD8^+^ T cells compared to NC (Fig. 5c), suggesting that PCMTD2 functions as a CUL5 adaptor protein negatively regulating the differentiation and activation of effector CD8^+^ T cells. To further confirm the importance of PCMTD2 in CUL5-mediated regulation of CD8^+^ T cell effector functions, we generated CUL5-PCMTD2 double KO (DKO). Both DKO showed significantly increased GZMB expression in CD8^+^ T cells and percentage of INFg-positive CD8^+^ T cells compared to NC ones, whereas it did not induce further CD8 activation compared to either CUL5 or PCMTD2 single KO (Supplementary Fig. 5c), supporting that CUL5 acts largely depending on PCMTD2 to regulate CD8^+^ T cell effector function. Of note, ASB6, which is a validated CUL5 binding protein^74^, was readily detected in CD8^+^ T cells (Supplementary Fig. 6a), but not enriched in the CUL5 co-IP samples, suggesting a possible selectivity of the CUL5/ELOC/ELOB complex for PCMTD2 in mouse CD8^+^ T cells. The lack of detection of WSB1, ASB3, SOCS3, or SOCS1 in the CUL5-HA IP samples may be due to low abundance of these proteins or selectivity in CD8^+^ T cells (Supplementary Fig. 6a). Cish binds to CUL5 in CD4^+^ T cells^41^ and was upregulated upon TCR stimulation in CD8^+^ T cells, but not enriched in CUL5-HA IP sample either, suggesting a possible differential utilization of substrate receptors between CD4^+^ and CD8^+^ T cells.

Several other proteins related to T cell activation and responses were also significantly enriched in the CUL5-HA IP samples (Fig. 5a), including the TCR complex molecules (CD3 subunits and TCRA), co-stimulator Cd28, IL2 receptor β subunit (IL2RB), IFITM3 (supporting resident memory T cell survival)^75^, TAX1BP1 (important for cell-cycle and mTORC signaling)^76^, and SKAP1 (important for LFA-1 adhesive activation)^77,78^. Because these proteins were upregulated in CUL5 KO CD8^+^ cells (Fig. 5e), among which the surface expression of TCR complex and IL2RB (Supplementary Fig. 6b) were further confirmed by flow cytometry, they may be direct substrates of the CUL5 E3 ligase complex through PCMTD2 for ubiquitination and subsequent degradation. Indeed, we found that PCMTD2 interacted with the intracellular domains of IL2RB and CD247 (Supplementary Fig. 5d-e) but not CD3e (Supplementary Fig. 5f) and that expression of CUL5 and PCMTD2 increased ubiquitination of the intracellular domains of IL2RB and CD247 (Supplementary Fig. 5g, h) and reduced their stability (Supplementary Fig. 5i, j) in HEK293 cells exogenously expressing these proteins. These data suggest that the CUL5 E3 ligase complex can target CD247 and IL2RB through its adaptor protein Pcmtd2. To detect ubiquitination changes affected by CUL5 KO in CD8 cells, we performed the tandem ubiquitin binding entities (TUBES) pull-down experiment using TCR-stimulated CUL5 KO and NC mouse CD8^+^ T cells followed by LC-MS analysis. The total protein lysates were used as internal controls. The contents of IL2RB, CD247, and CD28 pulled down by TUBES were reduced in CUL5 KO cells compared to those in the NC ones. By contrast, the contents of TCRA, CD3d, CD3e and CD3g pulled down by TUBES showed little differences (Supplementary Fig. 5k, Data 6). Moreover, comparison of the TUBES MS results with total protein MS and CUL5-IP MS results revealed only 8 proteins that show significant reduction in ubiquitination, enrichment in their protein content, and association with CUL5 (Supplementary Fig. 5k). These 8 proteins include two (IL2RB and CD147) that were validated biochemically, CD28, and several RNA binding proteins. Moreover, consistent with the importance of these molecules in T cell signaling, enrichment of a broad range of signaling pathways including the IL2/STAT5 signaling, AP1, CD8 TCR downstream signaling, IL12 signaling, cholesterol biosynthesis, TNF-alpha signaling via NF-kB, mTORC1 signaling, interferon gamma response, and cell cycle regulation pathways in CUL5 KO CD8^+^ T cells was revealed by pathway analysis of differentially expressed proteins based on the total protein MS data (Fig. 5d and Supplementary Fig. 6c). Increased ERK1/2 phosphorylation upon TCR stimulation (Fig. 5f) and STAT5 phosphorylation (Fig. 5g) in CUL5 KO CD8^+^ T cells further supports increased TCR downstream signaling and IL2/STAT5 signaling in CUL5 KO CD8^+^ T cells, which is consistent with the MS results.

Because CUL5 neddylation is required for the E3 ligase activity of the CUL5 complex, we tested two high-affinity neddylation inhibitors TAS4464 and MLN4924^79^ on CUL5 KO and control (NC) CD8^+^ T cells. Although there are other cullin proteins expressed in CD8 T cells (Supplementary Fig. 6d) whose activity also depends on neddylation^80^, TAS4464 increased GZMB and IFNg expression only in NC CD8^+^ T cells but not in CUL5 KO cells in both cytokine- (Fig. 5h) and TCR- (Fig. 5i) activation conditions, suggesting these two effector molecules are specifically regulated by CUL5, but not other cullin proteins in these cells. However, TAS4464 increased CD25 expression in both NC and CUL5 KO CD8^+^ T cells, suggesting other neddylated proteins may also negatively regulate CD25 in these cells (Supplementary Fig. 6e, f). MLN4924 showed similar GZMB and IFNg enhancing effects as TAS4464 did (Supplementary Fig. 6g, h), further confirming the importance of neddylation in CD8^+^ T cell regulation.

### CUL5 is a negative regulator of human CD8^+^ T cells

**Fig. 6:**
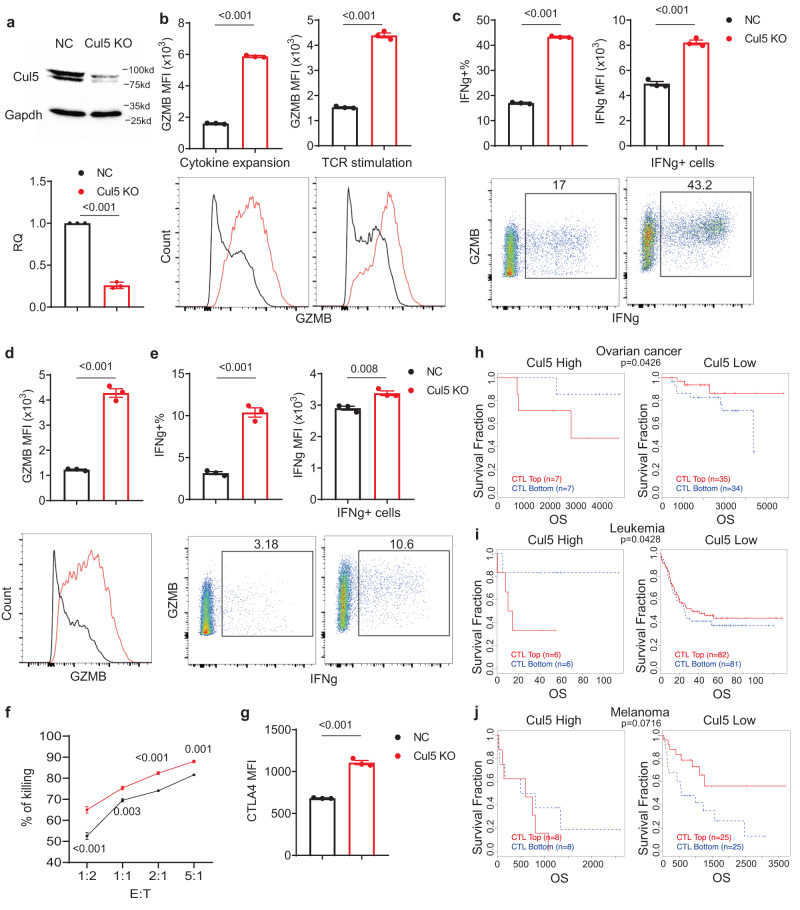
Characterization of CUL5 KO primary human CD8^+^ T cells. **a** Western blot analysis of CUL5 expression in primary human CD8^+^ T cells with NC (Black) or CUL5 KO (Red). Quantification of CUL5 protein levels normalized against GAPDH is shown (Bottom; *n* = 3; Two-sided unpaired t-test). **b** Flow cytometry analysis of GZMB expression in NC or CUL5 KO primary human CD8^+^ T cells before (Left) or after (Right) 6-hour anti-CD3 plus anti-CD28 stimulation. Data are shown in bar chart (Top) as mean ± SEM (Two-sided unpaired t-test, *n* = 3). **c** Flow cytometry analysis of IFNG^+^ cells and expression in NC (Black) or CUL5 KO (Red) primary human CD8^+^ T cells after 6-hour anti-CD3 plus anti-CD28 stimulation. Data are shown in bar chart (Top) as mean ± SEM (Two-sided unpaired t-test, *n* = 3). **d, e** Flow cytometry analysis of **d**, GZMB expression as MFI and **e**, IFNG expression as positive % in total (Left) and MFI in positive population (Right) in NC (Black) or CUL5 KO (Red) primary human CD8^+^ T cells transduced with CAR-CD19 after 6-hour co-culture with NALM6 B cell line. Data are shown in bar chart (Top) as mean ± SEM (Two-sided unpaired t-test, *n* = 3) and as representative histogram (**d**, Bottom) or scatter plot (**e**, Bottom).. **f** In vitro killing assay of NC (Black) or CUL5 KO (Red) CAR-CD19 primary human CD8^+^ T cells co-cultured with NALM6 B cell line overnight. E:T, T cell to NALM6 B cell ratios. The data presented as % of killing (mean ± SEM; Two-way Anova with Sidak correction, *n* = 3). **g** Flow cytometry analysis of CTLA4 expression in NC or CUL5 KO primary human CD8^+^ T cells 2-day post anti-CD3 plus anti-CD28 stimulation. Data are shown as mean ± SEM (Two-sided unpaired t-test, *n* = 3). **h–j** TIDE analyses of *CUL5* expression correlation with T cell dysfunction signatures and survival benefits in patients with **h**, ovarian cancer, **i**, leukemia and **j**, melanoma. (Left panels: patients with high *CUL5* expression; Right panels: patients with low *CUL5* expression). Datum points represent biological replicates.

To evaluate the human relevance and translational potential of our findings, we studied CUL5 changes at protein level in primary human CD8^+^ T cells. The total CUL5 protein detected by both flow cytometry (Supplementary Fig. 7a) and western blot (Supplementary Fig. 7b) was significantly upregulated upon TCR-stimulation. Neddylated CUL5 also increased (Supplementary Fig. 7b), consistent with the phenotype of mouse primary CD8^+^ T cells. We further performed CUL5 KO in primary human CD8^+^ T cells using the Cas9/sgRNA ribonucleoprotein electroporation approach. Three days post electroporation, CUL5 KO efficiency was evaluated by Western Blotting (Fig. 6a). Compared to the NC cells, CUL5 KO CD8^+^ T cells expressed significantly higher GZMB both before and after TCR-dependent activation (Fig. 6b). The percentage of IFNG^+^ cells and IFNG expression level are significantly higher in the CUL5 KO CD8^+^ T cells than the NC cells upon TCR-stimulation (Fig. 6c). There was no IFNG response or difference in CUL5 KO CD8^+^ T cells compared to the NC cells in the absence of TCR-stimulation (Supplementary Fig. 7c), suggesting CUL5 KO may not cause autoreactivity and tonic signaling in CD8^+^ T cells, thus minimizing the potential safety and accelerated exhaustion issues in clinical use. We further generated CD8^+^ chimeric antigen receptor (CAR)-T cells targeting CD19 by lentiviral transduction and sorted pure CAR-expressing cells by GFP reporter (Supplementary Fig. 7d). CUL5 KO CAR-T cells expressed significantly higher GZMB (Fig. 6d) and showed higher percentage of IFNG^+^ cells and IFNG expression level (Fig. 6e) after co-culturing with CD19-expressing NALM6 B cells, suggesting an increased CAR signaling activity in CUL5 KO CAR-T cells. Consistently, the CUL5 KO CAR-T cells not only showed significantly higher acute tumor cell killing ability of NALM6 B cells in culture than the NC cells (Fig. 6f) but also maintained the higher killing ability and viability after repeated stimulation (Supplementary Fig. 7e), demonstrating improved stemness potential, cytotoxic activity, and viability of CD8 + T cells upon CUL5 KO. Moreover, as shown in murine CD8^+^ T cells, CUL5 KO also resulted in significantly higher CTLA4 expression in human CD8^+^ T cells than the NC cells upon TCR re-stimulation suggesting a potential combinatory CAR-T therapy with CTLA4 blockade (Fig. 6g). To further access the clinical relevance of CUL5 in cancer, we applied the Tumor Immune Dysfunction and Exclusion (TIDE) analysis to examine the association of CUL5 expression^81^ with T cell dysfunction and disease outcomes. In multiple cancer types, including ovarian cancer (Fig. 6h), leukemia (Fig. 6i), and melanoma (Fig. 6j), high cytotoxic T cell score is associated with an overall survival benefit only when CUL5 expression is low, while high CUL5 expression abolishes and even reverses the beneficial effects of infiltrating cytotoxic T cells. Despite the limitations (low patient number and CUL5 expression not limited to CD8 T cells) of the TIDE analysis, the correlations are consistent with our data showing CUL5 plays important roles in CD8 cell activation and tumor killing functions.

## Discussion

**Fig. 7:**
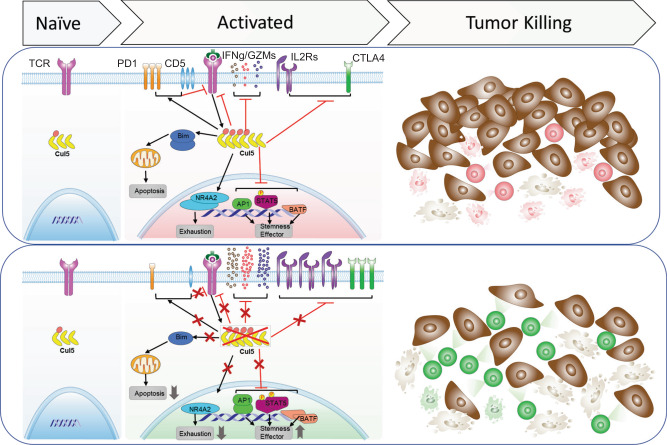
Illustration summary of CUL5 regulation of CD8^+^ T cell anti-tumor responses. The CUL5 protein and its neddylation are significantly upregulated upon TCR stimulation, which at least partially serves as a potential negative feedback regulator of TCR/IL2 signaling and anti-tumor effector activity in CD8^+^ T cells. CUL5 KO in CD8^+^ T cells significantly enhances their effector function, TCR and cytokine signaling, stemness, and survival accompanied with improved tumor control ability. The icons are created with BioRender.com.

In this study, we used a stepwise CRISPR/Cas9 screens in combination of proteomic analyses to identify a CUL5-E3 ligase complex as an important negative regulator of TCR/IL2 signaling and anti-tumor effector activity in CD8^+^ T cells. The CUL5 protein was significantly upregulated upon TCR stimulation, and its KO in CD8^+^ T cells significantly enhanced their effector function, TCR and cytokine signaling, stemness, and survival accompanied with improved tumor control ability in vitro and in vivo (Fig. 7). To date, different screening studies of CD8^+^ T cells have highlighted various candidates based on diverse in vitro and/or in vivo models and different readout criteria such as proliferation^21,23–25,27^, effector molecule (degranulation or cytokine) expression^21,22,61^ or subpopulation^26,82^. However, CUL5 was not identified as an enrichment hit in any of these studies, suggesting either enriched regulators in each screening are highly context-dependent or low-coverage in vivo screens tend to miss critical hits. Our stepwise screening strategy combining specific selection pressures with high confident coverages described in this study allowed identification of new hits including CUL5 that are important for signaling and anti-tumor activities of CD8^+^ T cells. Thus, this strategy provides a robust and cost-labor effective approach and can be readily adapted for other loss- or gain-of function screens with different selection pressures.

The signature gene sets in each cluster of perturb-seq in transferred CD8^+^ T cells in this study are overlapped with previous single cell RNA-seq (scRNA-seq) analysis on tumor-infiltrating CD8^+^ T cells^59,83^, which were used in our study to identify functional groups. One apparent difference from previous studies is that all tumor-infiltrating transferred CD8^+^ T cells in this study are specifically tumor-reactive with the same TCR clonal type while the sequenced tumor-infiltrating CD8^+^ T cells from patients are heterogeneous including both tumor-reactive ones and bystanders. For this reason, the clusters in our study are only a subset of previous studies. Some studies also have TCR-seq in parallel with scRNA-seq to track the dynamic differentiation of tumor-specific T cells. However, due to the high TCR diversity and limited tumor-infiltrating T cell sequencing ability, it is difficult to confidently conclude which clusters contain tumor-specific T cells derived from the same TCR clonal type. As the transferred T cells in our studies share the same known tumor-specific TCR, we demonstrated that tumor-reactive T cells can differentiate into stemness, effector, proliferating and exhaustion clusters, which will be very helpful to further investigate the dynamic changes between these clusters and critical genes regulating these dynamic changes in order to discover new targets of T cell-based immune therapies.

CUL5 E3 ubiquitin ligase-dependent protein degradation has been proposed as a mechanism by which the CIS/SOCS family proteins regulate T cell activation^39,40^. However, our study unexpectedly identified Pcmtd2, rather than other CIS/SOCS family proteins, as being associated with CUL5 in mouse CD8^+^ T cells. PCMTD2 has 79% amino acid sequence identity to its paralog PCMTD1^84^. Both the PCMTD molecules contain a Protein-L-Isoaspartate (D-aspartate) O-Methyltransferase domain and a SOCS-box domain. PCMTD1 can bind to the canonical methyltransferase cofactor S-adenosylmethionine (AdoMet) and CUL5/ELOB/ELOC complex, but does not appear to have the L-isoaspartyl methyltransferase activity^84^. PCMTD2, which also can bind to CUL5^73^, but not PCMTD1, was detected in our CUL5-HA co-IP MS analysis, suggesting PCMTD2 may be the dominant PCMTD isoform in CD8^+^ T cells. This may be due to low expression of PCMTD1 as it was undetectable in our total protein MS analysis of CD8^+^ T cells. The function of these PCMTD molecules is not known, even though Pcmtd1 was implicated in aging/stress-related protein repair^84^. The biochemical results and those showing that PCMTD2 KO in CD8^+^ T cells phenocopied CUL5 KO indicate that PCMTD2 is the substrate receptor for CUL5 in CD8^+^ T cells and hence reveal the first known in vivo function for the PCMTD proteins.

The CUL5-HA co-IP MS analysis also revealed an enrichment of the TCR/CD3 complex subunits, CD28, IL2RB, IFITM3 and TAX1BP1. TCR, CD28 co-stimulation and cytokines provide three signal traits that together induce optimal T cell activation. IL2 is critical for CD8^+^ T cell activation, proliferation and cytotoxic effector differentiation^49^ while IL15 is indispensable for the maintenance of memory CD8^+^ T cells^85^. Both cytokines signal through IL2RB. IFITM3 increases upon T cell activation and maintains T cell survival and effector functions in the lung tissue with influenza infection^75^. It is also constitutively expressed in tissue-resident memory T cells, suggesting its role in the memory survival. TAX1BP1 acts through autophagy induction in activated T cells to support mTORC activation and subsequent T cell proliferation^76^. Upon TCR-induced CD8^+^ T cell activation, the expression of CUL5 and its neddylation significantly increased, rendering CUL5 as a potential negative feedback regulator for TCR and IL2/IL15 signaling. Consistent with this idea, CUL5 KO in CD8^+^ T cells resulted in an upregulation of TCR and cytokine/JAK/STAT signaling, which may subsequently increase the activity and expression of T cell-activation related transcription factors, biogenesis and metabolism enzymes, and cytotoxic effector molecules, leading to enhanced anti-tumor activity. Beyond the enhanced effector functions, elevated JUN^86^ and BATF^87^ and decreased NR4A2 protein contents may explain increased exhaustion resistance^88,89^. Although some memory/stemness associated markers such as TCF7 and SLAMF6 were reduced upon CUL5 KO, CD62L^+^ population in activated T cells associated with memory phenotype increased, consistent with increased persistence and IL7R expression of effector cells in vivo and elevated live cell number post re-stimulation in vitro. These together suggest that CUL5 KO may not compromise stem-like differentiation.

It is interesting that two well-characterized immune checkpoint molecules, PD-1 and CTLA4, changed in opposite directions in CUL5 KO CD8^+^ T cells. Multiple pieces of evidence, including the lack of CTLA4 enrichment in CUL5 Co-IP (Fig. 5a) and TUBE-IP (Supplementary Data 6) as well as significantly increased CTLA4 transcript in CUL5 KO cells (Supplementary Fig. 4g), suggest that CTLA4 is unlikely the direct substrate of the CUL5 E3 ligase complex. It is also likely that CUL5 regulates other proteins besides TCR and IL2 signaling to contribute to the phenotypes of CUL5 KO in CD8 + T cells. PD-1 expression decreased, whereas CTLA4 increased, in the CUL5 KO CD8^+^ T cells compared to the control cells. The reduction in PD1 expression may reduce its immunosuppression and further facilitate activation of CD8^+^ T cells by CUL5 KO in tumors. The downregulation of PD1 may be due to the increase of SATB1 expression in the CUL5 KO CD8^+^ T cells, as SATB1 is a direct suppressor of PD1^90^ and negatively regulated by TGFβ^90,91^. The marked increase in CTLA4 expression in activated CUL5 KO CD8^+^ T cells, on the other hand, should suppress CUL5 KO CD8^+^ T cells by B7-expressing cells in tumor. Indeed, CTLA4/CUL5 DKO in CD8^+^ T cells showed further improved tumor control in vivo compared to CUL5 KO alone. This combinatory therapeutic potential of transferred CD8^+^ T cells with CUL5 KO may apply to other CUL5 KO-induced co-stimulatory molecules (ICOS and 4-1BB) by administration of their agonists or to co-inhibitory molecules (LAG3, TIGIT and TIM3) by their blockade. More importantly, the characteristics of CUL5 KO were translational from mouse to human CD8^+^ T cells. Namely, CUL5 KO enhanced TCR- or CAR-dependent T cell activation and improved cytokine-induced effector T cell differentiation in human CD8^+^ T cells. In addition to ACT, it is also possible that CUL5 is a potential immunotherapeutic target for small molecule intervention, as small molecule inhibitors for E3 ligases such as neddylation inhibitors are being actively investigated^92^. The observation that the neddylation inhibitor enhances CD8^+^ T cell activation and effector responses largely dependently of CUL5 supports these ideas and potential. Therefore, the CUL5-E3 ligase inhibitors may be tested as a single or combination with other ICIs, particularly anti-CTLA4 antibodies, giving high priority to the tumors in which *CUL5* expression shows positive correlations with the clinical CTL dysfunction signature. Since neddylation inhibitors are being tested clinically, attentions should be given to assess immunological responses of the treatment.

## Methods

### Cell lines and mice

HT-2, HEK293T, EL4, E.G7-OVA and NALM6 cell lines were purchased from ATCC. YUMM1.7 cell line was kindly provided by M.B. and was reported previously^93^. All cell lines were regularly tested for mycoplasma and confirmed to be negative. OT-I TCR transgenic mice (OT-I mice; Stock # 003831) and Constitutive Cas9-expressing mice (Cas9 mice; Stock # 026179) both with C57BL/6 background were purchased from Jackson lab. Cas9/OT-I mice were generated by crossing Cas9 and OT-I mice with genotyping following Jackson Lab protocols. 7-8-week-old female C57BL/6 N mice purchased from Envigo (Stock # 044) were used as E.G7-OVA tumor and adoptive T cell transfer recipients. Mice were co-housed in a specific-pathogen-free barrier facility, euthanized by CO_2_, and used in experiments under procedures approved by the Yale University Animal Care and Use Committee.

### Plasmids

The human Brie genome-wide CRISPR knockout pooled library in the pLentiCRISPRv2 one vector system (co-expressing spCas9 and sgRNA), with four sgRNAs per gene, was obtained from Addgene (Addgene # 73632, a gift from David Root and John Doench^54^) and prepared in the Yale Cancer Center Functional Genomics core). pMSCV-U6sgRNA(BbsI)-PGKpuro2ABFP was a gift from Sarah Teichmann (Addgene plasmid # 102796; http://n2t.net/addgene:102796; RRID:Addgene_102796). PGKpuro2ABFP was replaced by EF1a-core promoter-mScarlet gblock (IDT) to generate pMSCV-guide-EF1a-mScarlet vector compatible with Bio-Rad S3e sorting. For single cell perturb-seq, the original gRNA scaffold sequence was further replaced by a new scaffold sequence with 10x genomics compatible capture sequence 1 incorporated into the stem-loop to generate pMSCV-scguide-EF1a-mScarlet vector. Customized sub-library for bulk in vivo screening, with non-targeting control sgRNA sequences published previously and top ranked 6 sgRNA sequences per gene designed by CRISPick, was synthesized by Genscript and cloned into pMSCV-guide-EF1a-mScarlet. Customized sub-library for single cell in vivo screening, with 4 non-targeting control sgRNA sequences and top ranked 3 sgRNA sequences per gene selected from the above bulk sub-library, was synthesized by IDT and cloned into pMSCV-scguide-EF1a-mScarlet by NEB stable competent cells. The normal distribution and integrity of sub-libraries were confirmed by illumine next-generation-sequencing. Top two ranked murine Cd8a and *Cul5* sgRNAs (Supplementary Data 7) were designed by CRISPick, synthesized by IDT and cloned into pMSCV-guide-EF1a-mScarlet or pMSCV-scguide-EF1a-mScarlet vector by NEB stable competent cells. pSLCAR-CD19-BBz was a gift from Scott McComb (Addgene plasmid # 135992; http://n2t.net/addgene:135992; RRID:Addgene_135992). CUL5-HA overexpression retroviral plasmid was derived from in house MIGR-IRES-GFP vector. Mouse *Cul5* open-reading frame with HA tag at its C terminal was amplified by PCR. cDNA of TCR-activated mouse primary CD8^+^ T cells was used as PCR template. Primers were designed to add XhoI site at 5’ and HA sequence plus EcoRI site at 3’ of the PCR product (Supplementary Data 7). Backbone vector and PCR product were double digested by XhoI and EcoRI, and ligated by T4 ligation to get MIGR-CUL5-HA-IRES-GFP plasmid. Plasmids containing Myc-CUL5, HA-PCMTD2, GFP-CD247, GFP-CD3d, GFP-CD3e, GFP-CD3g and GFP-IL2RB overexpression plasmids were generated for transient expression in 293 T cells.

### Lentiviral and retroviral production

Lentiviruses with genome-wide murine CRISPR library or CAR-CD19 were produced in HEK293T cells transfected with library vector, pMD2.G (addgene, #12259) and psPAX2 (addgene, #12260). Retroviruses with sub-libraries or individual non-targeting or target gene sgRNAs were produced in HEK293T cells transfected with sublibrary/guide vector and pCL-Eco (addgene, #12371). Viral soups from 24-hour and 48-hour post-transfection were harvested, combined, filtered, aliquoted and saved in −80 °C freezer until use. The titers of lentiviruses and retroviruses of each batch were determined by the transduction of HEK293T and NIH3T3 cells respectively.

### In vitro genome-wide CRISPR KO screening

HT-2 cells were transduced with the lentiviral CRISPR/Cas9 library at MOI of 0.3 with over 200x coverage per sgRNA. About 25% transduction efficiency was confirmed by three-day puromycin selection to make sure one type of sgRNA per cell as the majority (Supplementary Fig. 1a). After 3 days of selection with 0.6 µg/ml puromycin, 2×10^7^ transduced HT-2 cells were saved as input, 2×10^7^ were cultured for 21 days either in 100 ng/ml IL2 medium (IL2) or in IL2 medium containing 120 pg/ml TGFβ1 (IL2 + TGF). For each condition, genomic DNA from 2×10^7^ cells was extracted by DNeasy blood & tissue kit (Qiagen, Cat.69504) and sgRNA cassettes were PCR amplified for illumina NGS^54^.

### In vivo bulk CRISPR KO screening

3×10^6^ EG.7-OVA cells were s.c. inoculated into 7-8 weeks old C56BL/6 N female mice. Tumor sizes were monitored every three days by caliper with volume calculation as v = d^2^xD/2. When tumors reached 0.5 cm^3^, untouched CD8^+^ T cells were isolated from the spleens of male and female Cas9/OT-I mice by CD8^+^ T cell isolation kit (MACS, Cat.130-104-075), and immediately stimulated by anti-mouse CD3/28 beads (Thermo Scientific, Cat.11456D) at 1:1 ratio for 24 h, followed by retroviral sub-library transduction at MOI of 0.2. Transduced CD8^+^ T cells were further expanded in IL2/7/15 medium for 3 days to ensure genome-editing completed. GFP/mScarlet double positive cells were then sorted on a Bio-Rad S3e sorter. 2×10^6^ cells were saved as input and 2×10^6^ sorted T cells per mouse were i.v. transferred into a total of four sub-lethally irradiated (4 Gy) EG.7-OVA tumor-bearing mice. 12 days post T cell transfer, GFP^+^ T cells were isolated and enriched from tumors, spleens and tumor-draining lymph nodes of recipient mice by sorting. Genomic DNA from sorted cells were extracted by Qiagen DNeasy blood & tissue kit. sgRNA cassettes were PCR amplified for illumina NGS.

### PCR of sgRNA cassettes for NGS

For all PCR products, a stagger P5-read1 forward primer mixture was used to increase NGS reading diversity, and different index-included P7-read2 reverse primers were used for PCR of individual replicate samples before pooled NGS. Primer sequences are shown in Supplementary Data 7. PCR reaction was set up according to NGS protocol of NEBNext Ultra^TM^ II Q5 Master Mix (NEB, Cat.M0544S). PCR products were purified by AMPure XP beads (Beckman Coulter, Cat. A63880) at 1:1 volume ratio. Purified PCR products were quantified by TapeStation (D1000 ScreenTape assay) before loading for NGS.

### In vivo single cell CRISPR KO screening

Tumor inoculation and sub-library transduced T cell transfer followed the same procedures of in vivo bulk CRISPR KO screening. 7 days post T cell transfer, live transferred tumor-infiltrating CD8^+^ T cells as CD8a^+^GFP^+^ T cells were sorted and washed once in cold 1x PBS containing 0.04% BSA prior to resuspending in cold 1x PBS containing 0.04% BSA at 1×10^6^ cells/ml concentration. Cells were then processed by Yale Center for Genome Analysis to generate single-cell RNA library and sgRNA library separately according to the instruction of 10x genomics 3’ V2 single cell RNA sequencing kit with sgRNA feature. Libraries were sequenced by illumina NGS.

### In vivo gene KO validation

1×10^6^ sorted NC or *Cul5* sgRNAs (Supplementary Data 7) transduced Cas9/OT-I T cells per mouse were i.v. transferred into sub-lethally irradiated (4 Gy) EG.7-OVA tumor-bearing mice in total of 4-6 mice per group when average tumor sizes reached around 1 cm^3^. Tumor sizes were monitored every two days post T cell transfer. CTLA4 KO was achieved by nucleofection of ribonucleoprotein (RNP) complex containing Cas9 protein (IDT, Cat.1081058) and Ctla4 sgRNA (Supplementary Data 7) into naïve CD8^+^ T cells before activation. At the endpoint, tumors and TDLNs were isolated to make single cell suspension for immediate flow cytometry staining or in vitro re-stimulation by PMA/ionomycin prior to flow cytometry staining. Metastasis into LN was defined as abnormally enlarged inguinal LN well separated from tumor mass.

### Tissue processes and transferred CD8^+^ T cell isolation

EG.7-OVA tumors were resected and minced into 1-2 mm pieces, followed by 20 min digestion at 37 °C in HBSS buffer containing Mg^2+^, Ca^2+^, HEPES, 2% FBS, collagenase, DNase I with constant shaking at 60 rpm speed. Digestion was stopped by 10 mM ETDA PBS solution and pass through 70 µm strainer. Remaining tumor pieces were further smashed with 3 ml syringe plunger and washed through the strainer. For CD8^+^ T cell sorting, tumor single cell suspension in 1xPBS was layered on top of Ficol-plus and centrifuged at 1000 g x 20 min without braking at room temperature. Buffy coat in which live CD8 T cells were enriched was collected and washed in 1xPBS. Spleens and TDLNs were directly smashed with 3 ml syringe plunger through 70 µm strainers. Red blood cell lysis buffer was further applied for smashed splenocytes to remove red blood cells. Single cell suspensions from different tissues were stained with PE-anti-mouse CD8a. GFP^+^ CD8a^+^ cells were sorted by Bio-Rad S3e sorter.

### In vitro CD8^+^ T cell stimulation and cancer cell killing assay

Following retroviral transduction of MACS purified Cas9/OT-I T cells as described above, GFP^+^mScarlet^+^ cells were sorted and expanded in culture medium containing 5 ng/ml hIL2 (R&D, Cat.202-IL-010), 2.5 ng/ml mIL7 (Peprotech, Cat.217-17) and 25 ng/ml mIL15 (Peprotech, Cat.500-P173) for 3-5 days. For flow cytometry detection of phosphorylated proteins, activation and effector markers, T cells at 1×10^6^/ml were kept in culture withdraw of cytokines for 8 h before stimulated with 1 µg/ml plate-coated anti-mouse CD3e (Biolegend, Cat.100340) and 1 µg/ml soluble anti-mouse CD28 (Biolegend, Cat.102116) for different time points with or without addition of transporter block (Thermo Scientific, Cat.00-4980-03). For some experiments, 250 nM TAS4464 (Selleckchem, Cat.S8849) was added for the inhibition of neddylation. Supernatants at different time points without the addition of transporter block were collected and kept in −80 °C freezer for cytokine ELISA detection later. For cancer cell killing assay, EL4 cells pre-stained with CFSE (Thermo Scientific, Cat.C34554) and EG.7-OVA cells pre-stained with CellTrace violet (Thermo Scientific, Cat.C34557) according to instruction were 1:1 mixed and seeded in U-bottom 96-well plate with 1×10^5^ total cells per well. T cells with different effector to target ratio were added and kept in culture overnight. Counting beads were added to calculate the absolute number of live CFSE^+^ EL4 cells and violet dye^+^ EG.7-OVA cells by flow cytometry. In some experiments, T cells and EL4/EG.7-OVA cells were co-cultured for 6 h with the addition of transporter block to detect antigen-specific cytotoxic T cell activation by flow cytometry.

### CUL5/PCMTD2 single and double KO in primary mouse CD8^+^ T cells

MACS-purified CD8^+^ T cells from spleens of WT mice were cultured in 5 ng/ml mIL7 overnight. Then PCMTD2 knockout of CD8^+^ T cells was achieved by CRISPR-Cas9. In brief, pre-designed crRNAs (IDT) for mouse CUL5 and PCMTD2 (Supplementary Data 7) or non-targeting control was annealed with tracrRNA (IDT, Cat.1072532) to form guide RNA, which then was mixed with Cas9 protein to generate ribonucleoprotein (RNP) complex. 1×10^6^ CD8^+^ T cells were re-suspended in P3 buffer (Lonza) containing RNP complex and enhancer DNA (IDT, Cat.1075915) and under electroporation with CM-137 program of 4D-nucleofactor X Unit (Lonza). Electroporated T cells recovered overnight in IL7 medium were then activated by anti-mouse CD3/CD28 beads (at 1:1 ratio for two days and expanded in culture medium containing 5 ng/ml hIL2, 2.5 ng/ml mIL7 and 25 ng/ml mIL15 for 2 days before experiments.

### RT-qPCR

RNA was extracted from cells by RNeasy plus mini kit (Qiagen, Cat.74034) and quantified by nanodrop. cDNA was then synthesized by iScript Reverse Transcription Supermix (Bio-Rad, Cat.1708841) for RT-qPCR from Bio-Rad. qPCR reaction was set up by using SsoAdvanced Universal SYBR Green Supermix (Bio-Rad, Cat.1725270) and run on CFX96 Real-Time PCR Detection System (Bio-Rad). Primers of individual genes were selected from PrimerBank as shown in Supplementary Data 6 and synthesized by IDT. The specificity of qPCR reaction was confirmed by melting curve. Relative quantification of individual genes was normalized to Actb.

### ELISA

Supernatants of in vitro stimulated CD8^+^ T cells without secretion blocking were harvested at 16-hour post stimulation, and stored in the −80 °C freezer before test. Mouse IFNg ELISA (Thermo Scientific, Cat.KMC4021) were performed according to the instruction. In brief, capture antibodies were coated in 96-well plate overnight in 4 °C. Cytokine standards and sample supernatants were then added, followed by the addition of detection antibodies and secondary antibody-HRP conjugation. After extensive washes between each step, TMB was added for 20 min at room temperature in dark. After stopping the reaction, absorbance at 450 nm was detected. IFNg concentrations were then calculated based on the standard curves.

### Flow cytometry

Cells were washed once in 1x cold PBS and resuspended in 50 µl 1xPBS containing anti-CD16/32 (BD, Cat.553142) and fixable aqua live/dead dye (Thermo Scientific, Cat.L34957) for 10 min on ice. Without wash, 50 µl surface antibody/tetramer mixture in 1xPBS was added for 15 min on ice, followed by 1x cold PBS wash twice. For intracellular cytokine, CTLA4, CUL5 and pERK1/2 staining, cells were fixed in 4% PFA for 20 min at room temperature and washed in 1x permeable buffer (Thermo Scientific, Cat.00-833356). 50 µl intracellular antibody mixture in 1x permeable buffer was added for 30 min at room temperature, followed by 1x permeable buffer twice. 50 µl anti-rabbit-PE or -Alexa Fluor 350 secondary antibody in 1x permeable buffer was added for 30 min at room temperature, followed by 1x permeable buffer twice. For intracellular pSTAT5 staining, cells were fixed in 4% PFA for 20 min at room temperature and washed in 1x PBS, followed by permeabilization in pre-colded methanol for 20 min on ice. After 1xPBS wash, 50 µl anti-pSTAT5-APC in 1xPBS was added for 30 min at room temperature, followed by 1xPBS wash twice. After final wash, cells were resuspended in 1x PBS and detected by BD LSR II. Antibodies used for flow cytometry are as follows:

anti-mouse: Biolegend: CD3-Pacific Blue (Cat.155611), CD8a-PE-Cy7 (Cat.100721), CD62L-BV605 (Cat.104437), CD11c-Pacific blue (Cat.117321), NK1.1-APC (Cat.108709), CD19-PE-Cy7 (Cat.115519), CD122-APC (Cat.105911), CD127-APC-Cy7 (Cat.135039), GZMB-APC (Cat.372203), IFNg-APC-Cy7 (Cat.505849), TNF-PE-Cy7 (Cat.506323), IL2-PB (Cat.503820), CD25-APC-Cy7 (Cat.101917), CD5-PB (Cat.100641), ICOS-APC (Cat.107711), PD1-PE-Cy7 (Cat.135215), CTLA4-APC (Cat.106309), CD62L-PE-Cy7 (Cat.104417), Vb5-PB (Cat.139515), Va2-PE (Cat.127807); Thermo Scientific: CD137-PB (Cat.48-1371-82), F4/80-APC(Cat.17-4801-82), CD4-BUV395(Cat.363-0042-80), Foxp3-PE-CY7(Cat.25-5773-80); BD: CD8-BUV395(Cat.563786), CD107a-APC (Cat.560646), pSTAT5-Alexa 647 (Cat.562076), anti-human (Biolegend): GZMB-APC (Cat.372203), IFNg-PE (Cat.502508), CTLA4-APC (Cat.369611), anti-rabbit: IgG-PE (Thermo Scientific, Cat. P-2771MP), IgG-Alexa Fluor 350 (Thermo Scientific, Cat.A-11069), anti-pERK1/2 (Cell Signaling Technology, Cat. 9101), Rabbit polyclonal IgG anti-CUL5 (Thermo Scientific, Cat.A302-173A); SIINFEKL-H-2K(b) tetramer-BV421 (NIH Tetramer Core Facility, Cat.53995)

### Proteomics analysis and co-IP for mass spectrometry

For proteomics measurements, 1×10^6^ cytokine expanded CD8^+^ T cells with or without CUL5 KO were either immediately harvested as T0 or continuously cultured in medium without cytokines for 8 h. Then cytokine deprived CD8^+^ T cells were harvested either immediately as T8 or after 16 h stimulation with 1 µg/ml plate-coated anti-CD3 and 1 µg/ml soluble anti-CD28 as T16. Harvested cells were washed in cold PBS for 3 times, snap frozen in liquid nitrogen and stored in −80 °C freezer. Cell pellets were lysed by the lysis buffer 10 M urea containing the cOmplete™ protease inhibitor cocktail (Roche, #11697498001). The cell tubes were ultrasonicated at 4 °C for two cycle (1 min per cycle) using a VialTweeter device (Hielscher-Ultrasound Technology)^94,95^, and then centrifuged at 20,000 x g for 1 h to remove the insoluble material. For the supernatant protein mixture, the reduction and alkylation were conducted with 10 mM Dithiothreitol (DTT) for 1 h at 56 °C and then 20 mM iodoacetamide (IAA) in dark for 45 min at room temperature. The samples were diluted by 100 mM NH4HCO3 and digested with trypsin (Promega) at ratio of 1:20 (w/w) overnight at 37 °C. The digested peptides purification was performed on C18 column (MarocoSpin Columns, NEST Group INC) and 1 µg of the purified peptides was injected for mass spectrometry analysis.

For co-IP in triplicate, 1×10^7^ CD8^+^ T cells per replicate transduced with MIGR-IRES-GFP control vector or MIGR-CUL5-HA-IRES-GFP CUL5-HA overexpression vector were stimulated in 1ug/ml anti-CD3 coated plate plus 1ug/ml soluble anti-CD28 for 12 h. 10 µM MG-132 was added for the last 8 h before harvesting cells. Harvested cells were washed in cold PBS for 3 times and immediately resuspended in IP lysis buffer (Thermo Scientific, Cat.87787) with proteinase/phosphatase inhibitor cocktail on ice for 10 min. After a high-speed centrifuge, supernatant was transferred into a new tube, followed by overnight incubation with anti-HA antibody (Biolegend, Cat.901515). Protein G dynabeads (Thermo Scientific, Cat.10003D) were then added with rotation at room temperature for 1 hour. Dynabeads containing bound protein complex were sequentially washed with cold IP lysis buffer without proteinase/phosphatase inhibitor cocktail 4x, and wash buffer without detergent 2x on magnetic stand. Protein complex was eluted twice by elution buffer containing 0.5 M ammonium hydroxide and 0.5 mM EDTA. Combined elutes were snap frozen in liquid nitrogen and stored in −80 °C freezer. The elution was dried with SpeedVac, and then resolved with 50 μL 6 M urea for reduction and alkylation. Other proteomic sample preparation steps were identical with the above cell sample protocol.

The samples were measured by data-independent acquisition (DIA) mass spectrometry method as described previously^96–98^. The Orbitrap Fusion Lumos Tribrid mass spectrometer (Thermo Scientific) instrument coupled to a nanoelectrospray ion source (NanoFlex, Thermo Scientific) and EASY-nLC 1200 systems (Thermo Scientific, San Jose, CA). A 120-min gradient was used for the data acquisition at the flow rate at 300 nL/min with the temperature controlled at 60 °C using a column oven (PRSO-V1, Sonation GmbH, Biberach, Germany). Each DIA-MS cycle consisted of one MS1 scan and 33 MS2 scans of variable isolated windows with 1 m/z overlapping between windows. The MS1 scan range was 350 – 1650 m/z and the MS1 resolution was 120,000 at m/z 200. The MS1 full scan AGC target value was set to be 2E6 and the maximum injection time was 100 ms. The MS2 resolution was set to 30,000 at m/z 200 with the MS2 scan range 200 – 1800 m/z and the normalized HCD collision energy was 28%. The MS2 AGC was set to be 1.5E6 and the maximum injection time was 50 ms. The default peptide charge state was set to 2. Both MS1 and MS2 spectra were recorded in profile mode. DIA-MS data analysis was performed using Spectronaut v15^99–101^ with “directDIA” by searching against the mouse SwissProt protein database. The oxidation at methionine was set as variable modification, whereas carbamidomethylation at cysteine was set as fixed modification. Both peptide and protein FDR cutoffs (Qvalue) were controlled below 1% by Spectronaut and the resulting quantitative data matrix were exported from Spectronaut. The sample-wise normalization was disabled for IP-MS experiments. All the other settings in Spectronaut were kept as Default.

### Tandem Ubiquitin Binding Entities (TUBES) IP for mass spectrometry

CUL5 KO and NC mouse CD8^+^ T cells were stimulated with flask coated anti-CD3 and soluble anti-CD28 for 16 h with 10 µM MG132 added in the last 8 h before harvesting. Then TUBES (LifeSensors, Cat. UM-0501M-1000) was used to pull down ubiquitinated proteins based on the instruction of the kit that later were measured by mass spectrometry as above.

### Human T cell culture and manipulation

CD8^+^ T cells were purified from human PBMCs by untouched human CD8^+^ T cell isolation kit (MACS, Cat.130-094-156) and cultured at 1×10^6^/ml in RPMI1640 medium containing 10% FBS, 5 ng/ml hIL7 (R&D, Cat.207-IL-005) and 50 ng/ml hIL15 (R&D, Cat.247-IL-005) overnight. Then CUL5 knockout of CD8^+^ T cells was achieved by CRISPR-Cas9. In brief, pre-designed crRNA (IDT) for human CUL5 (Supplementary Data 7) or non-targeting control was annealed with tracrRNA (IDT, Cat.1072532) to form guide RNA, which then was mixed with Cas9 protein to generate ribonucleoprotein (RNP) complex. 2×10^6^ CD8^+^ T cells were re-suspended in P3 buffer (Lonza) containing RNP complex and enhancer DNA (IDT, Cat.1075915) and under electroporation with EO-115 program of 4D-nucleofactor X Unit (Lonza). Electroporated T cells recovered overnight in IL7 and Il15 medium were then activated by anti-human CD3/CD28 beads (Thermo Scientific, Cat.11131D) at 1:1 ratio for two days. For CAR-T generation, one day post beads activation, T cells were transduced with lentiviruses containing CAR-CD19 by spinfection supplied with 8 µg/ml polybrene and 5 ng/ml hIL2. Activation beads were magnetically removed two days later and T cells at 5×10^5^/ml concentration were expanded in culture medium containing 5 ng/ml hIL2, 5 ng/ml hIL7 and 50 ng/ml hIL15. Fresh medium with cytokines was replaced every two days to keep T cell concentration lower than 2×10^6^/ml.

### Co-IP-Western Blot

For co-IP experiments carried out in HEK293T cells, cells were transfected with the indicated constructs for 24 h and MG132 (20 µM) was added to the culture medium 4hrs before collection. Cells were then lysed in a cell lysis buffer (50 mM HEPES, 150 mm NaCl, 1% Triton X-100, 0.5% NP40, 2 mM MgCl2, 2 mM EGTA, 10% Glycerol, protease inhibitor mixture (Roche), and phosphatase inhibitor mixture (Roche)). After centrifugation, the supernatants were immunoprecipitated by the indicated antibodies and then analyzed by western blotting. Antibodies used for the Co-IP experiments are anti-HA (Biolegend, 901513), anti-Myc (Proteintech, 16286-1-AP), and anti-GFP (Santa Cruz, sc-9996).

For co-IP of endogenous proteins in human primary CD8 cells, NC and CUL5 KO CD8^+^ T cells were prepared as described above and cultured with MG132 (20 µM) for 4hrs before collection. Immunoprecipitation was carried out as described above with anti-CUL5 antibody (Thermo Fisher A302-173A), followed by Western analysis using the same anti-CUL5 antibody and an -anti-PCMTD2 antibody (Thermo Fisher PA5-69557).

### Ubiquitination assay

The transfected cells were treated with MG132 (20 µM) for a duration of 6-8 h and subsequently collected in a denaturing buffer (6 M guanidine-HCl, 0.1 M Na2HPO4/NaH2PO4 pH 7.5, 10 mM imidazole). The lysates were then incubated with Ni-nitrilotriacetic acid (Ni-NTA) agarose beads for 3 h. This was followed by four washes with the denaturing buffer and two additional washes with a low-salt buffer (25 mM Tris-HCl at pH 6.8, 20 mM imidazole). The beads were then eluted by boiling in the SDS sample buffer in the presence of 200 mM imidazole. After centrifugation, the supernatants were subjected to analysis via western blotting.

### Western Blotting

WT mouse naïve CD8^+^ T cells were activation by anti-CD3/CD28 beads at 1:1 ratio for 0 or 48 h. Human CD8^+^ T cells were activated by anti-CD3/CD28 beads at 1:1 ratio for two days and NC or CUL5 KO ones were further expanded in 5 ng/ml hIL2, 5 ng/ml hIL7 and 50 ng/ml hIL15. Cells were then pelleted and washed twice with cold 1xPBS, followed by direct lysis in 2x SDS loading buffer (Thermo scientific, Cat.NP0007). Rabbit polyclonal IgG anti-CUL5 (Thermo Scientific, Cat.A302-173A) was used to detect mouse or human CUL5 expression. Rabbit polyclonal IgG anti-PCMTD2 (Thermo Scientific, Cat. PA5-69557) was used to detect mouse PCMTD2 expression. Mouse monoclonal IgG anti-GAPDH (Proteintech, Cat.60004-1-Ig), rabbit monoclonal IgG anti-Hsp90 (Proteintech, Cat. 13171-1-AP) and rabbit monoclonal IgG anti-Histone H3 (Cell Signaling Technology, Cat. 4499) were used as internal controls.

### Human CD8^+^ T cell activation in vitro

Cytokine expanded CD8^+^ T cells were stimulated in 1 µg/ml plate-coated anti-CD3 and 1 µg/ml soluble anti-CD28. CAR-T cells were stimulated with NALM B cells (ATCC, Cat.CRL-3273) at 1:2 ratio. Stimulated CD8^+^ T cells or CAR-T cells were cultured for 6 h with the addition of transporter inhibitor prior to flow cytometry staining.

### Human CAR-T cell in vitro killing

T cells expressing CAR-CD19 were sorted based on GFP reporter. Sorted CAR-T cells were co-cultured with CellTrace violet stained NALM B cells at different effector to target (E:T) ratio overnight. Violet^+^ live NALM B cells were detected by flow cytometry with counting beads added to calculate absolute number. Killing % =(live B cells in B cell culture alone – live B cells in co-culture)/live B cells in B cell culture alone. For chronic and repeated killing assay, CAR-T cells were stimulated every two to three days in 6-well plate coated with 1ug/ml anti-CD3 before harvesting for overnight killing setup. Remaining CAR-T cells were transferred into new 6-well plate for next round repeated activation and killing.

### Bioinformatics analysis

#### Bulk CRISPR KO screening analysis

Initial quality check was performed using the FastQC program and sequence adapters were trimmed using the Cutadapt tool. Genome-scale and enriched sub-library scale CRISPR/Cas9 KO screening performed using MAGeCK version 0.5.9.2^102^. MAGeCK uses a maximum likelihood-based estimation (MLE) to measure Z-scores for each gene from the log2 fold changes of each sgRNAs in a robust approach. We rank our sgRNAs/genes based on robust ranking aggregation (RRA) and p-values. For bulk in vivo screening, four replicates in each group (Tumor, Spleen and Tumor-draining lymph node and Input) expression were averaged and positive RRA scores were used for the identification of top-ranked genes.

#### In vivo single-cell CRISPR KO screening analysis

Seurat 4.0 was used to process single-cell sequencing data. In the quality control (QC) analysis, poor quality cells with nFeature_RNA < 600, nCount_RNA < 1200, log10(Gene Per UMI) < 0.8 and Mitochondrial gene percentage over 10% were excluded. Genes with zero count numbers were removed. In addition, TCR genes were removed to prevent clustering bias caused by the contribution of variable V(D)J transcripts in major variable components. Cell cycle markers from Tirosh et al., 2015^103^ is loaded with Seurat and cell-cycle scores for G2M, S or G1 phase were quantified and assigned to each cell as the metadata. In addition, the sgRNA data was added into corresponding Seurat objects as the metadata. The cells with more than 1 sgRNA or without sgRNA were excluded. The feature-barcode matrix was first normalized and scaled with default settings in Seurat. Then the 3000 top variable genes were identified, which served as the input to principal component analysis (PCA) for dimensionality reduction. We applied Louvain algorithm for clustering and retained the 20 leading principal components as an input for further visualization. The UMAP embedding was used to visualize the single cells on a two-dimensional space with a perplexity of 100. Cell clusters were annotated with hall marker genes. Differentially expressed gene (DEG) analysis among different cell groups were based on the non-parametric Wilcoxon rank sum test with logfc.threshold=0.25 and min.pct =0.1. Functional analyses of sgRNA perturbations were evaluated by DEG analysis of sgRNA specific groups. Enrichr^104,105^ was then used to perform signaling pathway enrichment analysis with The Molecular Signatures Pathway Database (MsigDB_Hallmark_2020) to discover enriched biological pathways based on the list of identified DEGs.

#### Proteomics data statistical analysis

The two-sided Student’s t-test was used to find the differentially abundant proteins, Protein groups with *p* < 0.01 and a fold-change > 1.8 were reported as significant in total protein MS while *p* < 0.05 and a fold-change >1.5 in co-IP MS. The R software was used for the data virtualization with the packages ggplot2 (boxplot), scatterplot (volcano plots), pheatmap (correlation), corrplot (correlation), factoextra (PCA). The GO enrichment analysis was performed by Metascape^106^ with default Express Analysis. All scripts for bioinformatics analysis will be available upon request.

### Statistics and reproducibility

Animals were grouped unblinded, but investigators were blinded for most of the qualification experiments. No data were excluded from analysis. Minimal group sizes for tumor progression studies were determined by using power calculations with the DSS Researcher’s Tookit with an α of 0.05 and power of 0.8. Statistical methods are described in the figure legends and statistical analysis was done with GraphPad Prism V10. Replicates used in statistical analysis are biological replicates.

### Reporting summary

Further information on research design is available in the Nature Portfolio Reporting Summary linked to this article.

### Supplementary information

Supplementary Information

Description of Additional Supplementary Files

Supplementary Data 1

Supplementary Data 2

Supplementary Data 3

Supplementary Data 4

Supplementary Data 5

Supplementary Data 6

Supplementary Data 7

Reporting Summary

### Source data

Source data

## Data Availability

The single cell sequencing data has been deposited to GEO. The accession number is GSE213921. All the mass spectrometry datasets and processed results have been deposited to the ProteomeXchange Consortium via the PRIDE partner repository with the dataset identifier PXD036793. Source data are provided with this article. Source data are provided with this paper.
