# Supplementary material for: The CUL5 E3 ligase complex negatively regulates central signaling pathways in CD8+ T cells
Source: Nat Commun. 2024 Jan 19;15:603. doi: 10.1038/s41467-024-44885-0 (PMC10798966; doi:10.1038/s41467-024-44885-0)
Supplement: Supplementary file 1 — Supplementary Information [file 41467_2024_44885_MOESM1_ESM.pdf]

SUPPLEMENTAL INFORMATION

The Cul5 E3 Ligase Complex Negatively Regulates Central Signaling Pathways in CD8+ T Cells

Xiaofeng Liao[1,2†], Wenxue Li[2], Hongyue Zhou[1,2], Barani Kumar Rajendran[1,2], Ao Li[2], Jingjing Ren[4], Yi Luan[1,2], David A. Calderwood[2], Benjamin Turk[2], Wenwen Tang[1,2], Yansheng Liu[2,3], Dianqing Wu[1,2,5]

Vascular Biology and Therapeutic Program[1], Department of Pharmacology[2], Yale Cancer Research Institute[3], Department of Dermatology[4], Yale Cancer Center[5], Yale University School of Medicine, New Haven CT 06520.

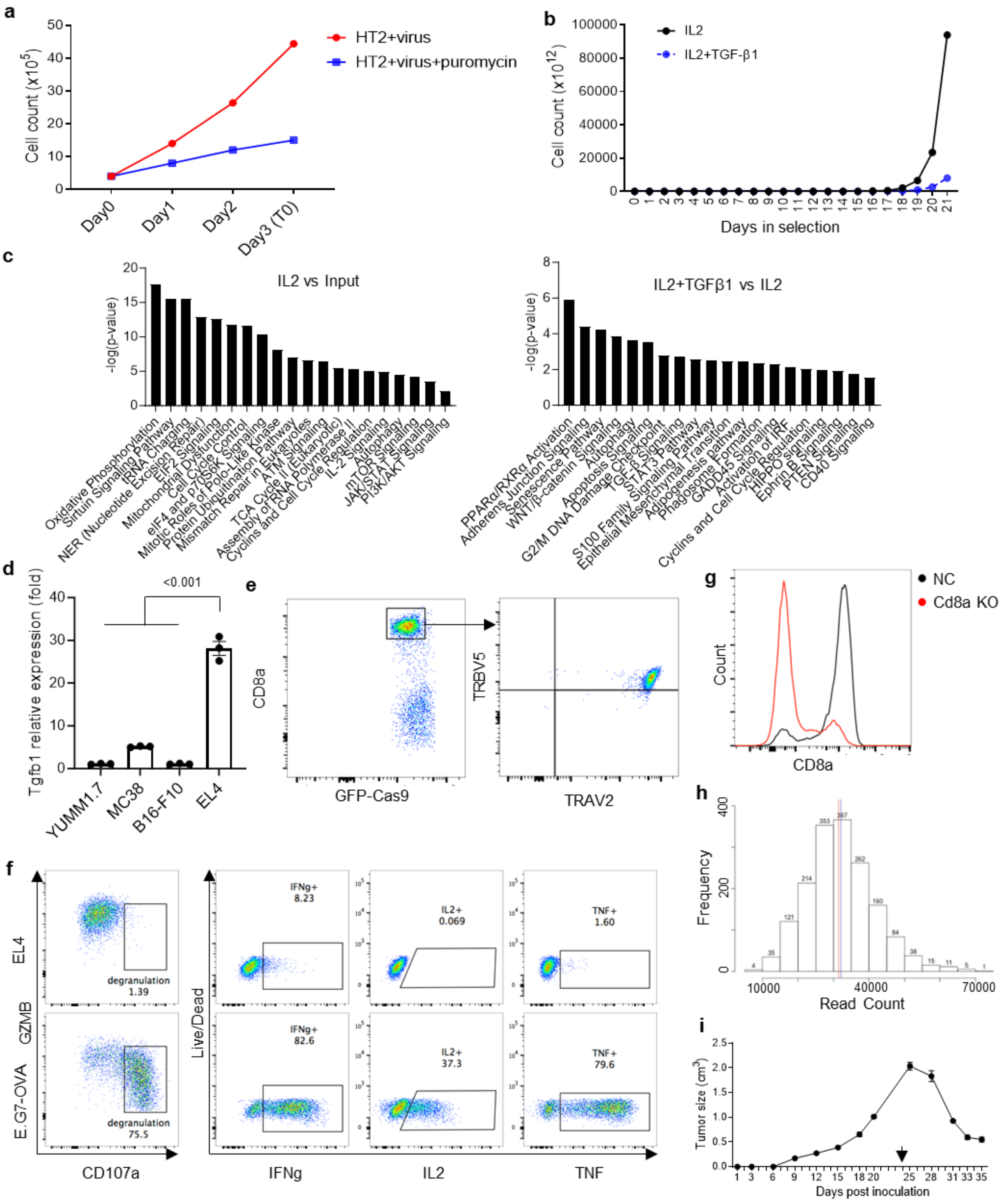

Supplementary Fig. 1 Bulk *in vitro* and *in vivo* CRISPR KO screens identify genes enhancing anti-tumor activity of CD8[+] T cells

a, *In vitro* growth curve of HT-2 cells with (Blue) or without (Red) puromycin selection post library lentiviral transduction. **b,** *In vitro* growth curve of puromycin-selected HT-2 cells transduced with genome-wide CRISPR KO library in IL2 (Black) or IL2 plus TGF-β1 (Blue) culture condition. **c,** Ingenuity pathway analysis of significantly depleted genes of IL2 vs input and enriched genes of IL2+TGFβ1 vs IL2. Complete lists of the pathways are show in Supplementary Table 1. **d,** qPCR of Tgf-β1 mRNA level in different tumor cell lines. Data are shown as mean + SEM (one way ANOVA without corrections, n=3). **e,** Flow cytometry analysis of Cas9/OT-I T cells regarding GFP, TRBV5 and TRAV2 expressions. **f,** Flow cytometry analysis of the expression of activation markers (GZMB, CD107a, IFNg, IL2 and TNF) in Cas9/OT-I T cells post co-culture with EL4 (Top) or E.G7-OVA (Bottom) cells. **g,** Cd8a surface expression of Cas9/OT-I cells transduced with Cd8a-targeting sgRNA (Red) compared to NC (Black) detected by flow cytometry. **h,** Normal distribution of sgRNAs in the sublibrary showing mean (Red) and median (Blue) values close to each other. **i,** Growth curve of tumors from E.G7-OVA cells inoculated s.c. into C57BL/6N mice (n=4). Data are shown as mean $\pm$ SEM. Black arrow indicates the time of sub-lethal irradiation followed by immediate adoptive transfer of Cas9/OT-I cells transduced with sgRNA sub-library.

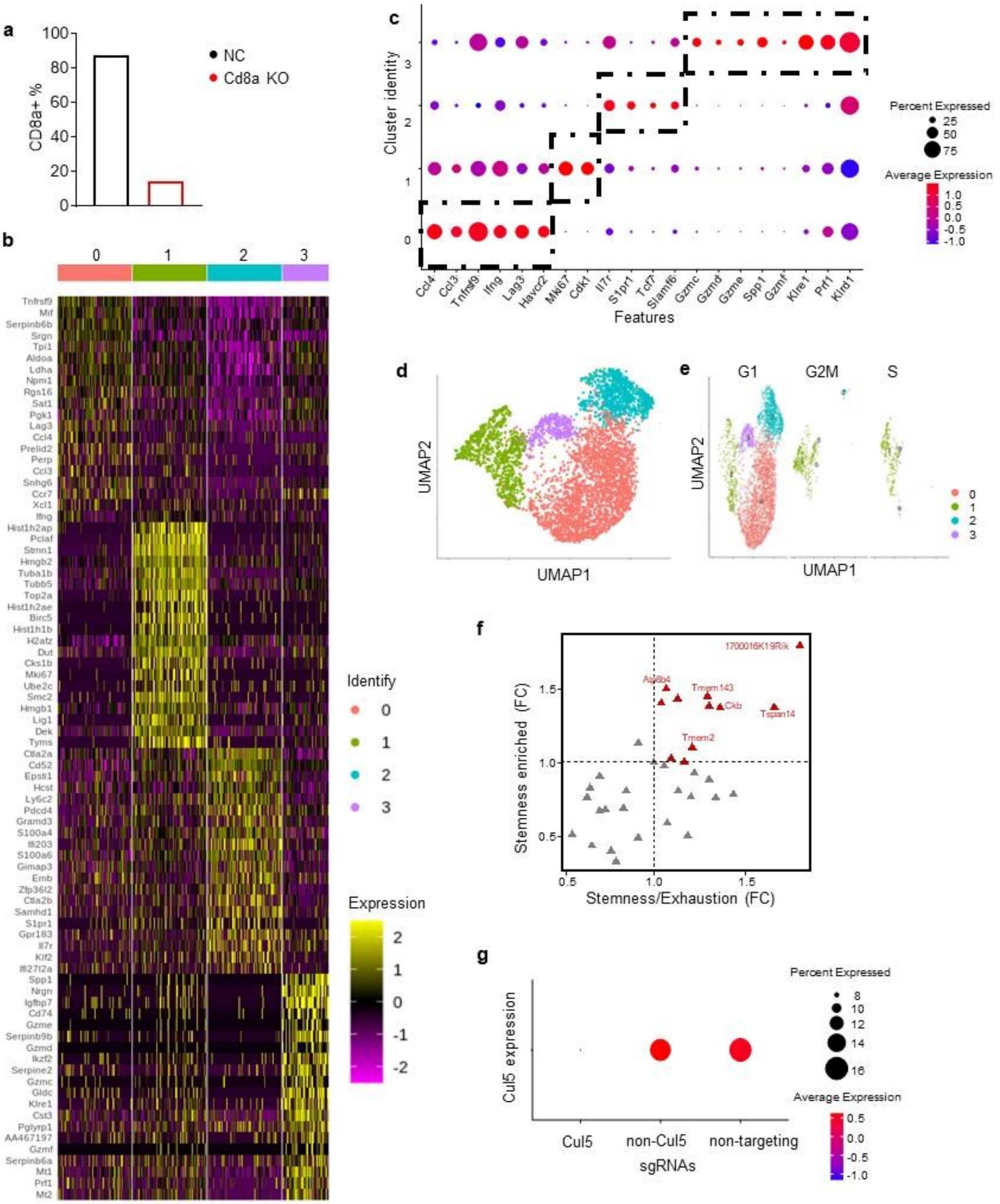

Supplementary Fig. 2 *In vivo* single-cell CRISPR KO screen identifies genes enhancing anti-tumor activity of CD8⁺ T cells

a, Flow cytometry analysis of CD8a expression on Cas9/OT-I T cells transduced with single-cell compatible non-targeting- (Black) or CD8a-sgRNA-containing (Red) retroviruses. **b,** Heatmap of

differentially expressed genes in each cluster of tumor-infiltrating Cas9/OT-I T cells derived from single-cell RNA sequencing data. **c,** Dotplot of the expression of key DEGs in each cluster used to annotate T cell subtypes. **d,** UMAP projection of transferred tumor-infiltrating Cas9/OT-I cells transduced with the second sgRNA sub-library with 10x scaffold, colored by DEG clusters. **e,** UMAP visualization of cell clusters in cell cycle phases (G1, G2M and S). **f,** At gene level normalized to non-targeting control, sgRNA ratio of stemness cluster to the exhausted cluster as X axis and sgRNA enrichment in stemness cluster compared to input as Y axis. **g,** Dotplot of Cul5 gene expression in transferred tumor-infiltrating Cas9/OT-I cells expressing Cul5, non-targeting and the other sgRNAs.

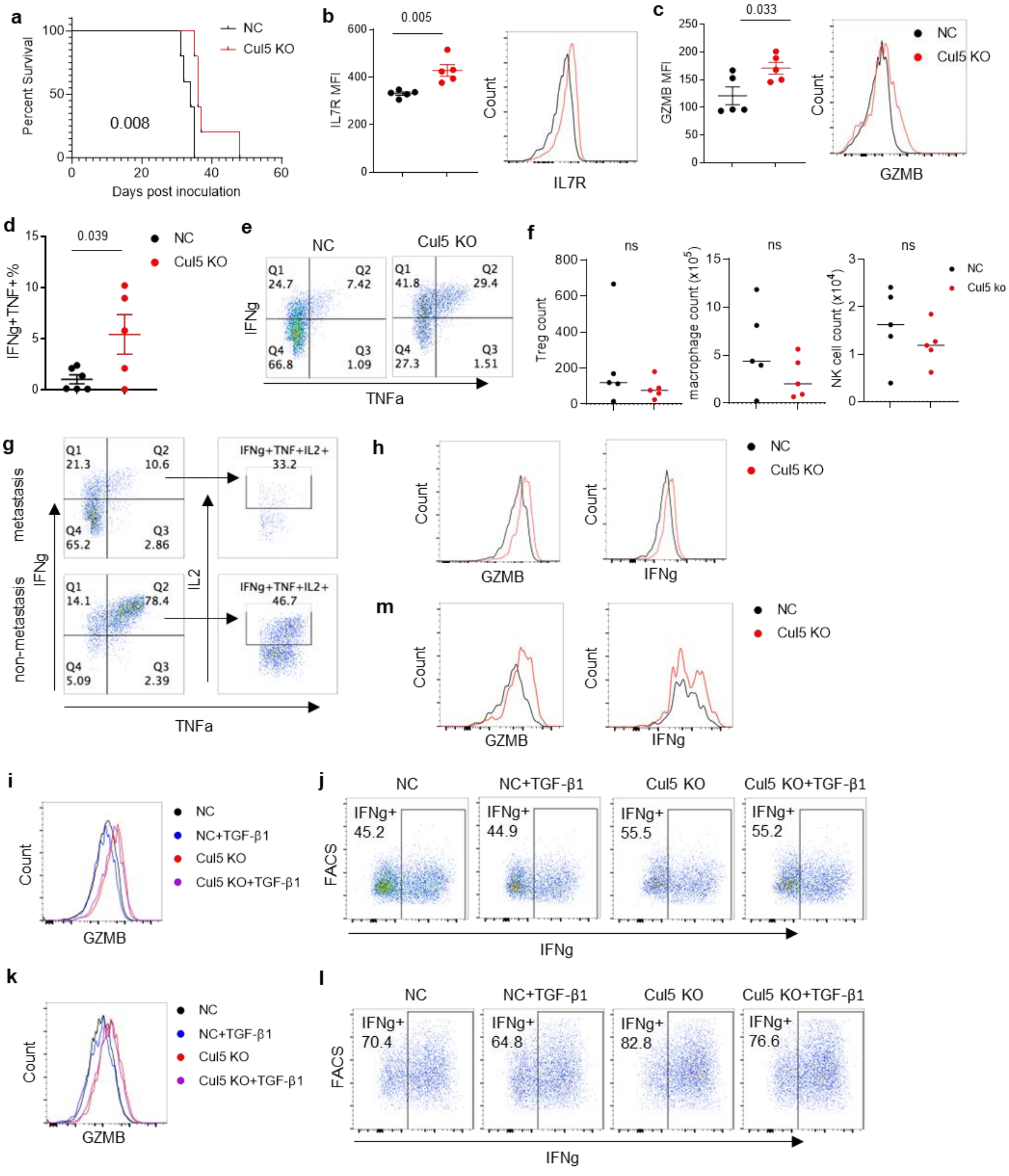

Supplementary Fig. 3 Cul5 KO enhances anti-tumor responses in primary CD8⁺ T cells *in vivo*
a, Prolonged survival curve monitoring of E.G7-OVA tumor-bearing C57BL/6N mice post adoptive transfer of Cul5 KO (Red) or NC (Black) Cas9/OT-I cells. *p<0.05 by Gehan-Breslow-Wilcoxon test (n=5 mice per group). **b,c,** Flow cytometry analysis of **b,** CD127 and **c,** Granzyme B expression of transferred TDLN-infiltrating Cas9/OT-I cells with Cul5 or non-targeting KO. Left panel in each plot is a scatter plot of median fluorescent intensity (MFI) for each marker. Right

panel is a representative histogram of each marker expression in Cul5 (Red) or NC (Black) KO T cells. Data are shown as mean ± SEM (Two-sided unpaired t-test, n=5). **d,** Flow cytometry analysis of $IFNg^{+}TNF^{+}$ cell counts per unit tumor of transferred tumor-infiltrating Cas9/OT-I cells with Cul5 (Red) or NC (Black) KO post re-stimulation *in vitro*. Data are shown as mean ± SEM (Two-sided unpaired t-test; n=6 NC and 5 KO). **e,** Representative flow cytometry plots of Fig. 2e. **f,** Flow cytometry analysis of Treg (Left), macrophage (Middle) and NK (Right) cell counts per tumor at the endpoint. **g,** Representative flow cytometry plots of Fig. 2g. **h-l,** Representative flow cytometry plots of Fig. 3a-3e respectively. **m,** Representative flow cytometry plots of Fig. 3h. Each datum point represents a mouse.

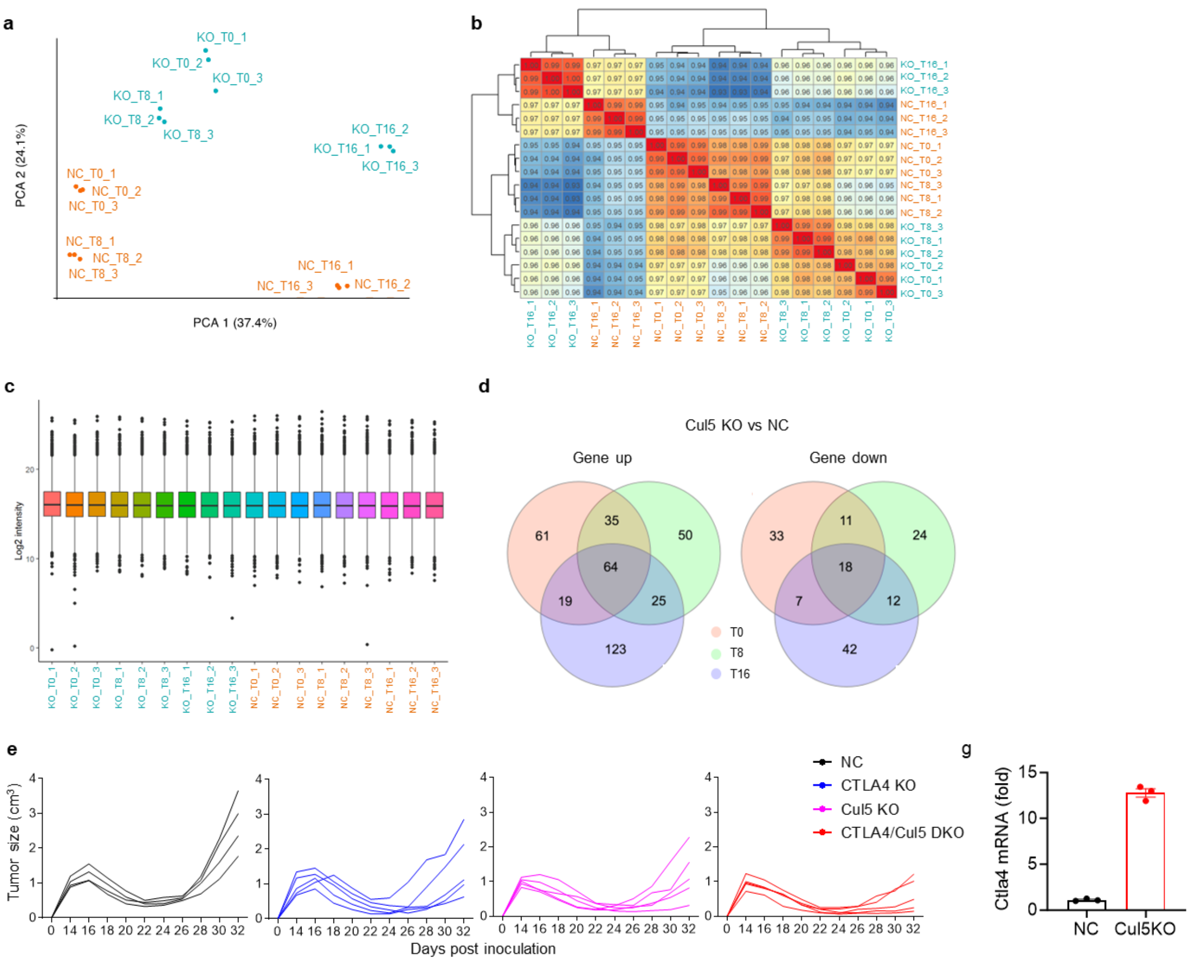

Supplementary Fig. 4 Cul5 KO causes proteomic alterations in primary CD8⁺ T cells

a, Principal component analysis of individual samples by the quantitative proteomics data using DIA-MS. **b,** Pearson correlation analysis between individual samples by proteomics data visualizing sample relationships. **c,** Normalized protein DIA-MS signals of individual samples by proteomics data. **d,** Venn diagram of proteins upregulated (Left) or downregulated (Right) in Cul5 KO primary CD8^{+} T cells compared to NC ones at T0, T8 and T16 conditions. **e,** Growth curve of tumors from E.G7-OVA cells inoculated s.c. into C57BL/6 mice. Data are shown as individual mouse. Black arrow indicates the time of sub-lethal irradiation followed by immediate adoptive transfer of Cas9/OT-I cells with NC (Black), Ctla4 (Blue), Cul5 (Pink) and Ctla4/Cul5 double (Red) KO. (n=4-5 mice per group). **g**, Determination of Ctla4 mRNA expression by RT-qPCT in cells described in Fig. 4f. Data are shown in means ± SEM ($p<0.001$ Unpaired Student t-test, n=3).

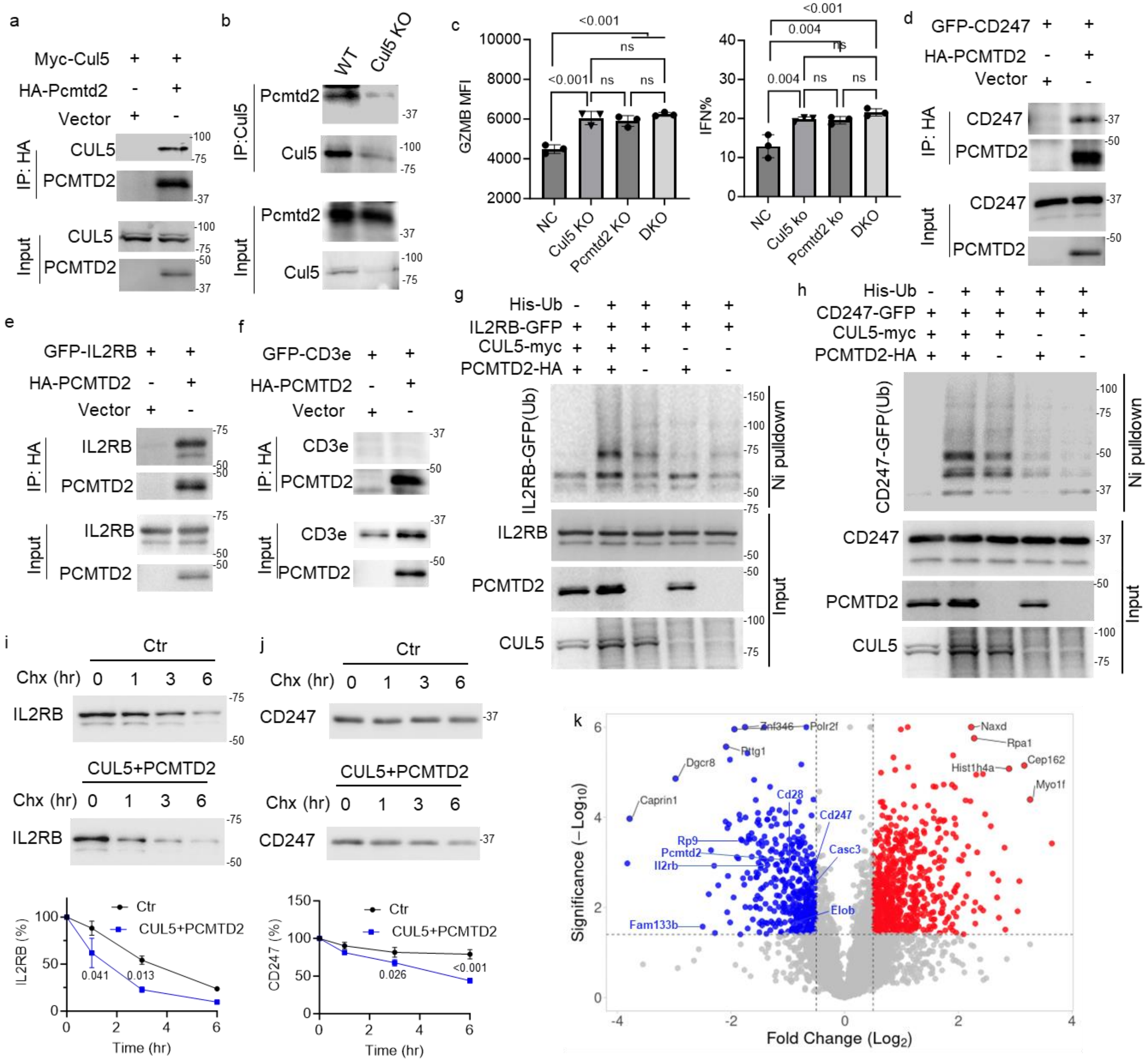

Supplementary Fig. 5 The Cul5 E3 complex targets TCR and IL2 signaling through Pcmtd2 in CD8+ T cells.

a, Western Blot detection of Myc-Cul5 after IP with HA-Pcmtd2. Myc-Cul5 and HA-Pcmtd2 were expressed in HEK293. **b,** Primary human CD8$^+$ T cells with NC or CUL5 KO were immunoprecipitated with anti-CUL5 followed by Western analysis. **c,** Flow cytometry analysis of GZMB expression and INFG percentage of NC, Cul5 KO, Pcmtd2 KO and DKO primary mouse CD8$^+$ T cells. Data are shown as mean ± SEM (Two-sided unpaired t-test, n=3). Datum points

represent biological replicates. **d,e,f,** Western Blot detection of the C-terminal domains of CD247, IL2RB, and CD3e fused with GFP after IP with HA-PCMTD2. These proteins were expressed in HEK293 cells. **g,h**, Ubiquitination of IL2RB and CD247 was detected after pulldown of His-Ub by Ni-NTA beads from HEK293 cells co-expressing His-ubiquitin (His-Ub), CUL5, PCMTD2, and the C-terminal domain of IL2RB or CD247 fused with GFP. **i,j**, Stability of IL2RB and CD247 proteins was examined by Western analysis of HEK293 cells expressing IL2RB or CD247 C-terminal domains fused with GFP (Ctr) or together with PCMTD2 and CUL5. Data are shown in means ± SEM (Two-Way Anova; n=3). **i,j.** Expression of of CUL5 and PCMTD2 promotes degradation of the C-terminal IL2BR and CD247 in HEK293. Cycloheximide (Chx) was added at 24 hours after transfected. Representative Western blots are shown. Data are shown as mean ± SEM (Two-way Anova; n=3). **k**, Volcano plot of normalized TUBES-MS results of CUL5 KO CD8 cells vs those of NC cells. The labels of the proteins that are showed significant ($p<0.05$, log2fold change >0.66) enrichments in both total protein and CUL5-IP MS analyses are highlighted in blue.

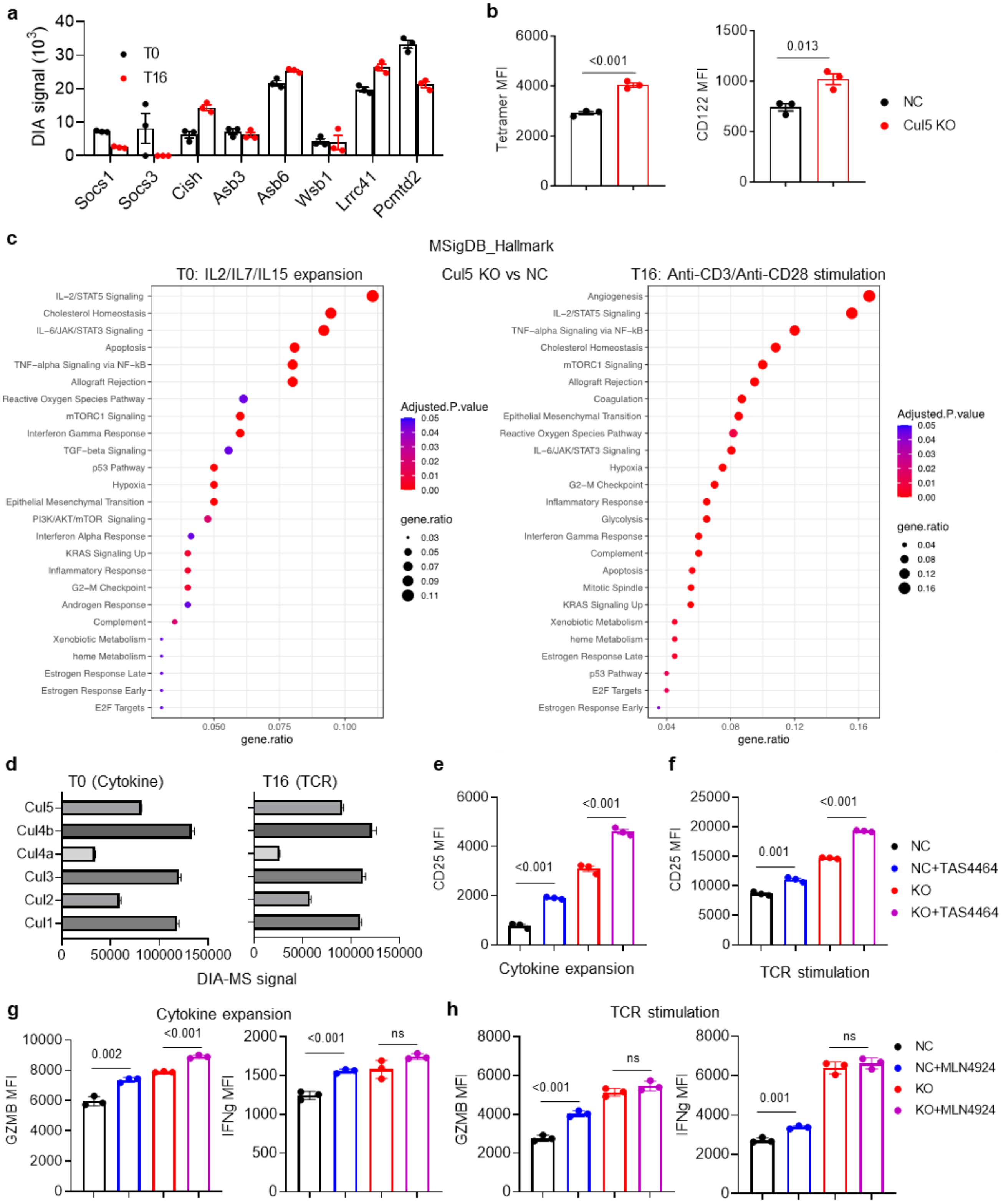
a
DIA signal (10^3)
T0
T16
Socs1
Socs3
Cish
Asb3
Asb6
Wsb1
Lrrc41
Pcmtd2
b
Tetramer MFI
<0.001
CD122 MFI
0.013
NC
Cul5 KO
c
MSigDB_Hallmark
T0: IL2/IL7/IL15 expansion
Cul5 KO vs NC
T16: Anti-CD3/Anti-CD28 stimulation
IL−2/STAT5 Signaling
Cholesterol Homeostasis
IL−6/JAK/STAT3 Signaling
Apoptosis
TNF−alpha Signaling via NF−kB
Allograft Rejection
Reactive Oxygen Species Pathway
mTORC1 Signaling
Interferon Gamma Response
TGF−beta Signaling
p53 Pathway
Hypoxia
Epithelial Mesenchymal Transition
PI3K/AKT/mTOR Signaling
Interferon Alpha Response
KRAS Signaling Up
Inflammatory Response
G2−M Checkpoint
Androgen Response
Complement
Xenobiotic Metabolism
heme Metabolism
Estrogen Response Late
Estrogen Response Early
E2F Targets
gene.ratio
Adjusted.P.value
Angiogenesis
Coagulation
Glycolysis
Mitotic Spindle
d
T0 (Cytokine)
T16 (TCR)
Cul5
Cul4b
Cul4a
Cul3
Cul2
Cul1
DIA-MS signal
e
CD25 MFI
<0.001
Cytokine expansion
f
0.001
TCR stimulation
NC
NC+TAS4464
KO
KO+TAS4464
g
Cytokine expansion
GZMB MFI
0.002
IFNg MFI
ns
h
TCR stimulation
NC+MLN4924
KO+MLN4924

Supplementary Fig. 6 Signaling pathway enrichment and neddylation inhibition in CD8$^+$ T cells with Cul5 KO

a, DIA-MS signals of different SOCS-box-containing proteins identified by the total protein MS analysis of NC mouse primary CD8$^+$ T cells under T0 (cytokine expansion, Black) and T16 (TCR stimulation, Red) conditions. **b,** Flow cytometry analysis of TCR complex (SIINFEKL-H-2K(b) tetramer$^+$, Left) and Il2rb (CD122, Right) expression of 16-hour anti-CD3/CD28-stimulated Cas9/OT-I cells with Cul5 (Red) or non-targeting (Black) KO. Data are shown as mean ± SEM (Two-sided unpaired t-test, n=3). **c,** MSigDB Hallmark signaling pathway enrichment in Cul5 KO primary CD8$^+$ T cells at T0 (Left) and T16 (Right) conditions. **d,** DIA-MS signals of different cullin proteins in NC mouse primary CD8$^+$ T cells under T0 (cytokine expansion) and T16 (TCR stimulation) conditions. **e,f,** Flow cytometry analysis of CD25 in NC (Black and Blue) or Cul5 KO (Red and Purple) primary CD8$^+$ T cells in 16-hour **e,** cytokine culture (IL2/IL7/IL15) or **f,** anti-CD3 plus anti-CD28 stimulation with (Blue and Purple) or without (Black and Red) 250nM TAS4464 addition. **g,h,** Flow cytometry analysis of GZMB and IFNg in NC (Black and Blue) or Cul5 KO (Red and Purple) primary CD8$^+$ T cells in 16-hour **g,** cytokine culture (IL2/IL7/IL15) or **h,** anti-CD3 plus anti-CD28 stimulation with (Blue and Purple) or without (Black and Red) 1uM MLN4924. Data are shown as mean ± SEM (Two-sided unpaired t-test, n=3). Datum points represent biological replicates.

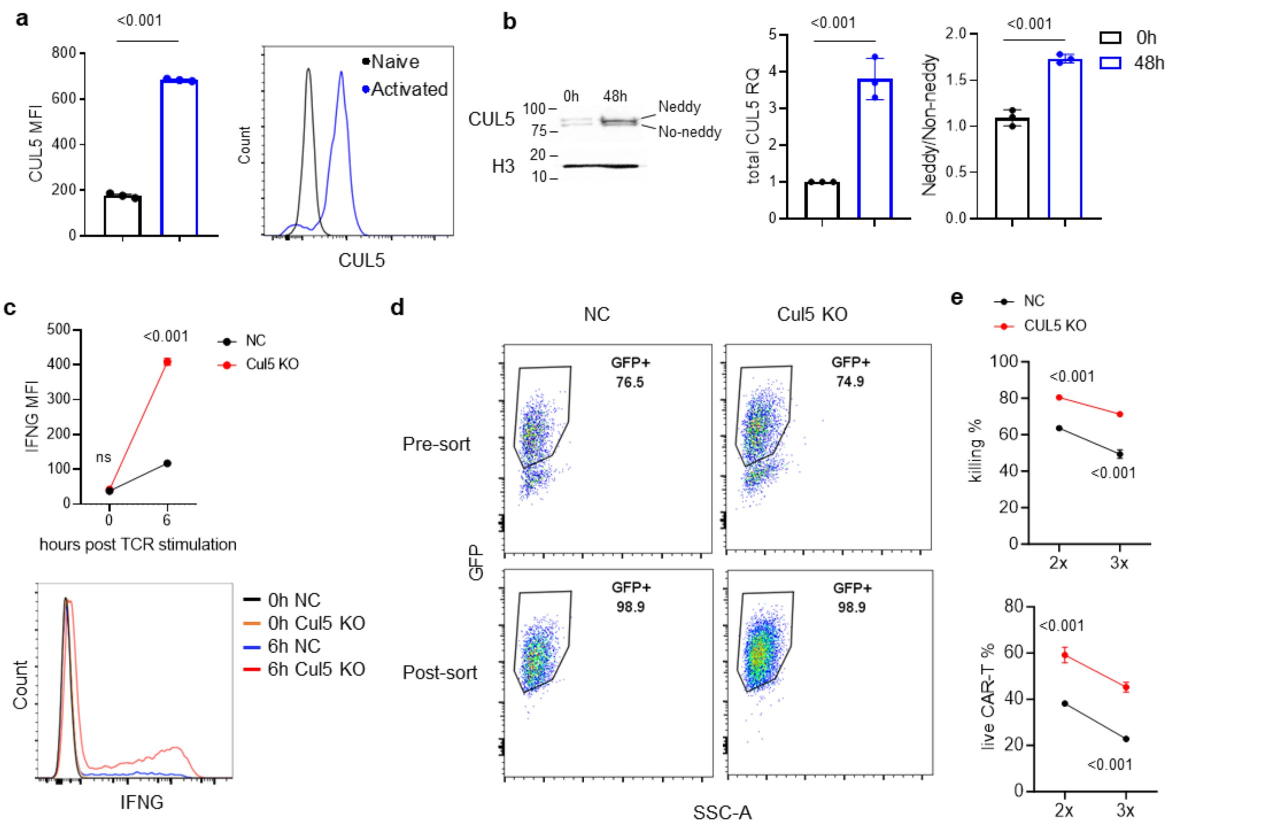

Supplementary Fig. 7 CUL5 KO in primary human CD8$^+$ T cells

a, Flow cytometry analysis of CUL5 expression in primary human CD8$^+$ T cells before (Black) or after (Blue) 2-day activation by anti-CD3/CD28 beads. Data are shown as mean ± SEM (Two-sided unpaired t-test, n=3). **b,** Western blot analysis of CUL5 expression in primary human CD8$^+$ T cells treated as in **a**. Histone H3 as an internal control. Bands were shown in the left panel. Relative quantity (RQ) of total CUL5 protein was normalized to Histone H3 in the middle panel. The ratio of neddylated to non-neddylated CUL5 was shown in the right panel. Data are shown as mean ± SEM (Two-sided unpaired t-test, n=3). **c,** Flow cytometry analysis of IFNG MFI in NC (Black) or CUL5 KO (Red) primary human CD8$^+$ T cells before and after 6-hour anti-CD3 plus anti-CD28 stimulation. Data are shown as mean ± SEM (Two-sided unpaired t-test; ns as not significant, n=3). **d,** Flow cytometry analysis of human CD8$^+$ CAR-CD19-T cells with NC or CUL5 KO pre- or post-sorting of GFP$^+$ population. **e,** *In vitro* killing assay of NC (Black) or Cul5 KO (Red) CAR-CD19 primary human CD8$^+$ T cells co-cultured with NALM6 B cell line overnight after 2x and 3x repeated TCR stimulation, with E:T=1:2. The data presented as % of killing (Top) and % of live CAR-T cells (Bottom) (mean ± SEM; two-sided multiple t-test, n=3). Datum points represent biological replicates.
