# Supplementary material for: The CUL5 E3 ligase complex negatively regulates central signaling pathways in CD8+ T cells
Source: Nat Commun. 2024 Jan 19;15:603. doi: 10.1038/s41467-024-44885-0 (PMC10798966; doi:10.1038/s41467-024-44885-0)
Supplement: Supplementary file 2 — Description of Additional Supplementary Files [file 41467_2024_44885_MOESM2_ESM.pdf]

Description of Additional Supplementary Files

File Name: Supplementary Data 1
Description: Bulk CRISPR screen data

File Name: Supplementary Data 2
Description: SgRNA sequences for in vivo single cell CRISPR screen

File Name: Supplementary Data 3
Description: Mass spectrometry analysis data of total proteins

File Name: Supplementary Data 4
Description: Differentially regulated proteins from mass spectrometry analysis of total

File Name: Supplementary Data 5
Description: Cul5-IP MS data

File Name: Supplementary Data 6
Description: TUBES-IP MS data

File Name: Supplementary Data 7
Description: Primer and sgRNA sequences
