# Supplementary material for: The CUL5 E3 ligase complex negatively regulates central signaling pathways in CD8+ T cells
Source: Nat Commun. 2024 Jan 19;15:603. doi: 10.1038/s41467-024-44885-0 (PMC10798966; doi:10.1038/s41467-024-44885-0)
Supplement: Supplementary file 10 — Reporting Summary [file 41467_2024_44885_MOESM10_ESM.pdf]

nature portfolio

Corresponding author(s): D. Wu

Last updated by author(s): Dec 13, 2023

Reporting Summary

Nature Portfolio wishes to improve the reproducibility of the work that we publish. This form provides structure for consistency and transparency in reporting. For further information on Nature Portfolio policies, see our Editorial Policies and the Editorial Policy Checklist.

Statistics

For all statistical analyses, confirm that the following items are present in the figure legend, table legend, main text, or Methods section.

n/a	Confirmed	
☐	☒	The exact sample size (*n*) for each experimental group/condition, given as a discrete number and unit of measurement
☐	☒	A statement on whether measurements were taken from distinct samples or whether the same sample was measured repeatedly
☐	☒	The statistical test(s) used AND whether they are one- or two-sided<br>*Only common tests should be described solely by name; describe more complex techniques in the Methods section.*
☐	☒	A description of all covariates tested
☐	☒	A description of any assumptions or corrections, such as tests of normality and adjustment for multiple comparisons
☐	☒	A full description of the statistical parameters including central tendency (e.g. means) or other basic estimates (e.g. regression coefficient) AND variation (e.g. standard deviation) or associated estimates of uncertainty (e.g. confidence intervals)
☐	☒	For null hypothesis testing, the test statistic (e.g. *F*, *t*, *r*) with confidence intervals, effect sizes, degrees of freedom and *P* value noted<br>*Give P values as exact values whenever suitable.*
☒	☐	For Bayesian analysis, information on the choice of priors and Markov chain Monte Carlo settings
☒	☐	For hierarchical and complex designs, identification of the appropriate level for tests and full reporting of outcomes
☐	☒	Estimates of effect sizes (e.g. Cohen's *d*, Pearson's *r*), indicating how they were calculated

Our web collection on statistics for biologists contains articles on many of the points above.

Software and code

Policy information about availability of computer code

Data collection	no software used for data collection
Data analysis	DIA-MS data analysis was performed using Spectronaut v15101-103 with “directDIA” by searching against the mouse SwissProt protein database.<br>The R software was used for the data virtualization with the packages ggplot2 (boxplot), scatterplot (volcano plots), pheatmap (correlation), corrplot (correlation), factoextra (PCA). The GO enrichment analysis was performed by Metascape1 with default Express Analysis.<br>Genome-scale and enriched sub-library scale CRISPR/Cas9 KO screening performed using MAGeCK version 0.5.9.2<br>Seurat 4.0 was used to process single-cell sequencing data.<br>Flowcytometry analysis was done with Flowjo V10.8<br>No custom software was used.

For manuscripts utilizing custom algorithms or software that are central to the research but not yet described in published literature, software must be made available to editors and reviewers. We strongly encourage code deposition in a community repository (e.g. GitHub). See the Nature Portfolio guidelines for submitting code & software for further information.

Data

Policy information about availability of data

All manuscripts must include a data availability statement. This statement should provide the following information, where applicable:

- Accession codes, unique identifiers, or web links for publicly available datasets
- A description of any restrictions on data availability
- For clinical datasets or third party data, please ensure that the statement adheres to our policy

The single-cell sequencing data has been deposited to GEO. The accession number is GSE213921. (To review the dataset please go to https://nam12.safelinks.protection.outlook.com/?url=https%3A%2F%2Fwww.ncbi.nlm.nih.gov%2Fgeo%2Fquery%2Facc.cgi%3Facc%3DGSE213921&data=05%7C01%7Cjingjing.ren%40yale.edu%7C138480356bee4bcf1dd908da9c98109b%7Cdd8cbebb21394df8b4114e3e87abeb5c%7C0%7C0%7C637994474170177851%7CUnknown%7CTWFpbGZsb3d8eyJWIjoiMC4wLjAwMDAiLCJQIjoiV2luMzIiLCJBTiI6Ik1haWwiLCJXVCI6Mn0%3D%7C3000%7C%7C%7C&sdata=s9wZzFbvvonyeGqr%2FzGP4J7MsuRpU6ReHqnUqd8a1%2BE%3D&reserved=0, with password: wfilwimmtbirpyl).

All the mass spectrometry datasets and processed results have been deposited to the ProteomeXchange Consortium via the PRIDE partner repository with the dataset identifier PXD036793 (To review the dataset please go to https://www.ebi.ac.uk/pride/login, and use the following login details: Username: reviewer_pxd036793@ebi.ac.uk, Password: EVCVbBJi).

Research involving human participants, their data, or biological material

Policy information about studies with human participants or human data. See also policy information about sex, gender (identity/presentation), and sexual orientation and race, ethnicity and racism.

Reporting on sex and gender	N/A
Reporting on race, ethnicity, or other socially relevant groupings	N/A
Population characteristics	N/A
Recruitment	N/A
Ethics oversight	N/A

Note that full information on the approval of the study protocol must also be provided in the manuscript.

Field-specific reporting

Please select the one below that is the best fit for your research. If you are not sure, read the appropriate sections before making your selection.

☒ Life sciences ☐ Behavioural & social sciences ☐ Ecological, evolutionary & environmental sciences

For a reference copy of the document with all sections, see nature.com/documents/nr-reporting-summary-flat.pdf

Life sciences study design

All studies must disclose on these points even when the disclosure is negative.

Sample size	Minimal group sizes for tumor progression studies were determined by using power calculations with the DSS Researcher's Tookit with an α of 0.05 and power of 0.8. All of non-animal studies were done at least in biological triplicates.
Data exclusions	No data were excluded from analysis.
Replication	Animal experiments were repeated at least twice and all of in vitro experiments were repeated at least three times.
Randomization	All mice were randomly assigned to experimental groups. Randomization was done in other experiments when possible.
Blinding	Animals were grouped unblinded, but investigators were blinded for most of the qualification experiments. For other experiments, samples were unblinded after data collections have been completed.

Reporting for specific materials, systems and methods

We require information from authors about some types of materials, experimental systems and methods used in many studies. Here, indicate whether each material, system or method listed is relevant to your study. If you are not sure if a list item applies to your research, read the appropriate section before selecting a response.

Materials & experimental systems

n/a	Involved in the study
☐	☒ Antibodies
☐	☒ Eukaryotic cell lines
☒	☐ Palaeontology and archaeology
☐	☒ Animals and other organisms
☒	☐ Clinical data
☒	☐ Dual use research of concern
☒	☐ Plants

Methods

n/a	Involved in the study
☒	☐ ChIP-seq
☐	☒ Flow cytometry
☒	☐ MRI-based neuroimaging

Antibodies

Antibodies used	anti-mouse CD3e (Biolegend, Cat.100340) 1:1000 dilution, anti-mouse CD28 (Biolegend, Cat.102116) 1:1000 dilution, anti-CD16/32 (BD, Cat.553142) 1:100 diution, Biolegend: CD3-Pacific Blue (Cat.155611), CD8a-PE-Cy7 (Cat.100721), CD62L-BV605 (Cat.104437), CD11c-Pacific blue (Cat.117321), NK1.1-APC (Cat.108709), CD19-PE-Cy7 (Cat.115519), CD122-APC (Cat.105911), CD127-APC-Cy7 (Cat.135039), GZMB-APC (Cat.372203), IFNg-APC-Cy7 (Cat.505849), TNF-PE-Cy7 (Cat.506323), IL2-PB (Cat.503820), CD25-APC-Cy7 (Cat.101917), CD5-PB (Cat.100641), ICOS-APC (Cat.107711), PD1-PE-Cy7 (Cat.135215), CTLA4-APC (Cat.106309), CD62L-PE-Cy7 (Cat.104417), Vb5-PB (Cat.139515), Va2-PE (Cat.127807); Thermo Scientific: CD137-PB (Cat.48-1371-82), F4/80-APC (Cat.17-4801-82), CD4-BUV395(Cat.363-0042-80), Foxp3-PE-CY7(Cat.25-5773-80); BD: CD8-BUV395(Cat.563786), CD107a-APC (Cat.560646), pSTAT5-Alexa 647 (Cat.562076), anti-human (Biolegend): GZMB-APC (Cat.372203), IFNg-PE (Cat.502508), CTLA4-APC (Cat.369611), anti-rabbit: IgG-PE (Thermo Scientific, Cat. P-2771MP), IgG-Alexa Fluor 350 (Thermo Scientific, Cat.A-11069) , anti-pERK1/2 (Cell Signaling Technology, Cat. 9101), Rabbit polyclonal IgG anti-Cul5 (Thermo Scientific, Cat.A302-173A); SIINFEKL-H-2K(b) tetramer-BV421 (NIH Tetramer Core Facility, Cat.53995). All flow cytometry antibodies with 1:100 dilution. All western antibodies with 1:1000 dilution.
Validation	No antibody validation was performed in this study. All of antibodies used in the study were chosen based on manufacturer's validation results or references on their websites.

Eukaryotic cell lines

Policy information about cell lines and Sex and Gender in Research

Cell line source(s)	HT-2, HEK293T, EL4, E.G7-OVA and NALM6 cell lines were purchased from ATCC
Authentication	No cell line validation was performed in this study. We relied on ATCC to provide us the correct cell lines.
Mycoplasma contamination	Cells have been routinely tested for mycoplasma and they were negative.
Commonly misidentified lines (See ICLAC register)	No

Animals and other research organisms

Policy information about studies involving animals; ARRIVE guidelines recommended for reporting animal research, and Sex and Gender in Research

Laboratory animals	8-12-week-old OT-I TCR transgenic mice (OT-I mice) and Constitutive Cas9-expressing mice (Cas9 mice) both with C57BL/6 background used as T cell donors were purchased from Jackson lab. 7-8-week-old C57BL/6N mice purchased from Envigo were used as E.G7-OVA tumor and adoptive T cell transfer recipients. Mice were housed under 12 hour dark/light cycle at 24 C without humidity control.
Wild animals	no wild animals were used
Reporting on sex	Sexes of mice used in experiments were specified in the Methods and figure legends.
Field-collected samples	No field collected samples were used
Ethics oversight	Mice were housed in specific-pathogen-free conditions with all procedures approved by the Yale University Animal Care and Use Committee.

Note that full information on the approval of the study protocol must also be provided in the manuscript.

Flow Cytometry

Plots

Confirm that:

- [x] The axis labels state the marker and fluorochrome used (e.g. CD4-FITC).
- [x] The axis scales are clearly visible. Include numbers along axes only for bottom left plot of group (a 'group' is an analysis of identical markers).
- [x] All plots are contour plots with outliers or pseudocolor plots.
- [x] A numerical value for number of cells or percentage (with statistics) is provided.

Methodology

Sample preparation	Cells were washed once in 1x cold PBS and resuspended in 50µl 1xPBS containing anti-CD16/32 (BD, Cat.553142) and fixable aqua live/dead dye (Thermo Scientific, Cat.L34957) for 10 minutes on ice. Without wash, 50µl surface antibody/tetramer mixture in 1xPBS was added for 15 minutes on ice, followed by 1x cold PBS wash twice. For intracellular cytokine, CTLA4 Cul5 and pERK1/2 staining, cells were fixed in 4% PFA for 20 minutes at room temperature and washed in 1x permeable buffer (Thermo Scientific, Cat.00-833356). 50µl intracellular antibody mixture in 1x permeable buffer was added for 30 minutes at room temperature, followed by 1x permeable buffer twice. 50µl anti-rabbit-PE or -Alexa Fluor 350 secondary antibody in 1x permeable buffer was added for 30 minutes at room temperature, followed by 1x permeable buffer twice. For intracellular pSTAT5 staining, cells were fixed in 4% PFA for 20 minutes at room temperature and washed in 1x PBS, followed by permeabilization in pre-colded methanol for 20 minutes on ice. After 1xPBS wash, 50µl anti-pSTAT5-APC in 1xPBS was added for 30 minutes at room temperature, followed by 1xPBS wash twice. After final wash, cells were resuspended in 1x PBS and
Instrument	BD LSR II
Software	Flow Jo Ver 10
Cell population abundance	N/A
Gating strategy	Gating examples are shown

- [x] Tick this box to confirm that a figure exemplifying the gating strategy is provided in the Supplementary Information.
